# Tissue factor cytoplasmic domain exacerbates post-infarct left ventricular remodeling via orchestrating cardiac inflammation and angiogenesis

**DOI:** 10.7150/thno.63354

**Published:** 2021-09-03

**Authors:** Suet Yen Chong, Olga Zharkova, Siti Maryam J.M. Yatim, Xiaoyuan Wang, Xiong Chang Lim, Chenyuan Huang, Chia Yee Tan, Jianming Jiang, Lei Ye, Michelle Siying Tan, Veronique Angeli, Henri H. Versteeg, Mieke Dewerchin, Peter Carmeliet, Carolyn S.P. Lam, Mark Y. Chan, Dominique P.V. de Kleijn, Jiong-Wei Wang

**Affiliations:** 1 Department of Surgery, Yong Loo Lin School of Medicine, National University of Singapore, Singapore, Singapore; 2 Cardiovascular Research Institute (CVRI), Cardiovascular Disease TRP, National University Heart Centre Singapore (NUHCS), Singapore, Singapore; 3 Department of Biochemistry, Yong Loo Lin School of Medicine, National University of Singapore, Singapore, Singapore; 4 National Heart Research Institute Singapore, National Heart Centre Singapore, Singapore.; 5 Immunology translational research program, Department of Microbiology and Immunology, Yong Loo Lin School of Medicine, National University of Singapore, Singapore,; 6 Immunology Program, Life Sciences Institute, National University of Singapore, Singapore; 7 Einthoven Laboratory for Experimental Vascular Medicine, Leiden University Medical Centre, The Netherlands; 8 Laboratory of Angiogenesis and Vascular Metabolism, Center for Cancer Biology (CCB), VIB, Leuven, Belgium.; 9 Laboratory of Angiogenesis and Vascular Metabolism, Department of Oncology and Leuven Cancer Institute (LKI), KU Leuven, Leuven, Belgium; 10 National Heart Centre Singapore (NHCS), Duke-NUS Graduate Medical School, Singapore, Singapore.; 11 Department of Cardiology, University Medical Center, Groningen, The Netherlands.; 12 Department of Medicine, Yong Loo Lin School of Medicine, Singapore, Singapore.; 13 Netherlands Heart Institute, Utrecht, the Netherlands.; 14 Department of Vascular Surgery, University Medical Center Utrecht, Utrecht, The Netherlands.; 15 Department of Physiology, Yong Loo Lin School of Medicine, National University of Singapore, Singapore, Singapore.; 16 Nanomedicine translational research programme, Centre for NanoMedicine, Yong Loo Lin School of Medicine, National University of Singapore, Singapore, Singapore.

**Keywords:** tissue factor cytoplasmic domain, myocardial infarction, inflammation, angiogenesis, adverse left ventricular remodeling

## Abstract

The coagulation protein tissue factor (TF) regulates inflammation and angiogenesis via its cytoplasmic domain in infection, cancer and diabetes. While TF is highly abundant in the heart and is implicated in cardiac pathology, the contribution of its cytoplasmic domain to post-infarct myocardial injury and adverse left ventricular (LV) remodeling remains unknown.

**Methods:** Myocardial infarction was induced in wild-type mice or mice lacking the TF cytoplasmic domain (TF∆CT) by occlusion of the left anterior descending coronary artery. Heart function was monitored with echocardiography. Heart tissue was collected at different time-points for histological, molecular and flow cytometry analysis.

**Results:** Compared with wild-type mice, TF∆CT had a higher survival rate during a 28-day follow-up after myocardial infarction. Among surviving mice, TF∆CT mice had better cardiac function and less LV remodeling than wild-type mice. The overall improvement of post-infarct cardiac performance in TF∆CT mice, as revealed by speckle-tracking strain analysis, was attributed to reduced myocardial deformation in the peri-infarct region. Histological analysis demonstrated that TF∆CT hearts had in the infarct area greater proliferation of myofibroblasts and better scar formation. Compared with wild-type hearts, infarcted TF∆CT hearts showed less infiltration of proinflammatory cells with concomitant lower expression of protease-activated receptor-1 (PAR1) - Rac1 axis. In particular, infarcted TF∆CT hearts displayed markedly lower ratios of inflammatory M1 macrophages and reparative M2 macrophages (M1/M2). *In vitro* experiment with primary macrophages demonstrated that deletion of the TF cytoplasmic domain inhibited macrophage polarization toward the M1 phenotype. Furthermore, infarcted TF∆CT hearts presented markedly higher peri-infarct vessel density associated with enhanced endothelial cell proliferation and higher expression of PAR2 and PAR2-associated pro-angiogenic pathway factors. Finally, the overall cardioprotective effects observed in TF∆CT mice could be abolished by subcutaneously infusing a cocktail of PAR1-activating peptide and PAR2-inhibiting peptide via osmotic minipumps.

**Conclusions:** Our findings demonstrate that the TF cytoplasmic domain exacerbates post-infarct cardiac injury and adverse LV remodeling via differential regulation of inflammation and angiogenesis. Targeted inhibition of the TF cytoplasmic domain-mediated intracellular signaling may ameliorate post-infarct LV remodeling without perturbing coagulation.

## Introduction

Globally, ischemic heart disease is a leading cause of death 1, with myocardial infarction (MI) being the most common complication. Following MI, the infarcted myocardium undergoes a dynamic remodeling process with early recruitment of phagocytes to clear necrotic cardiomyocytes (inflammatory phase) followed by proliferation of endothelial cells and fibroblasts to form a vascularized granulation tissue (proliferative phase) and finally formation of a collagen-rich scar (maturation phase) 2. Precise control of this complex process is critical for optimal post-MI cardiac repair and left ventricular (LV) remodeling. Overactive MI-induced inflammatory responses may be deleterious, with accentuation of myocardial extracellular matrix (ECM) degradation over collagen production and poor-quality cardiac repair with wall thinning and LV chamber dilation, culminating in heart failure or even cardiac rupture 3. Conversely, inadequate inflammation or ECM degradation with a vigorous and widespread fibrotic response may also result in suboptimal cardiac repair and a stiff and dysfunctional LV 2, 3. Neovascularization via angiogenesis can restore nutrient supply to ischemic myocardium, whereas impaired angiogenesis at the proliferative phase will retard cardiac repair and worsen adverse LV remodeling 4.

Tissue factor (TF) is a transmembrane protein with its extracellular domain forming a TF-VIIa(-Xa) coagulation protease complex that initiates the extrinsic coagulation cascade 5, 6. Most TF is present at the extravascular sites to provide a hemostatic barrier for blood vessels 6, however, TF is also abundantly expressed in human and murine hearts, with the highest levels in the LV myocardium 7. Low TF expression is found in the LV of patients experiencing hypertension, ventricular hypertrophy, or sepsis 7, 8. In mice, TF deficiency causes severe cardiac fibrosis and LV dysfunction due to spontaneous intramyocardial hemorrhage. Lack of the cytoplasmic domain of TF, however, does not affect cardiac development or function 9, 10. These findings demonstrate that the TF extracellular domain plays a pivotal role in cardiac homeostasis, whereas the TF cytoplasmic domain appears dispensable at steady state.

In fact, the TF cytoplasmic domain, consisting of 20 amino acids in mice, is not required for TF coagulation activity but mediates TF intracellular signaling pathways via the G-protein-coupled protease-activated receptors (PARs), in particular PAR1 and PAR2, in cancer metastasis, inflammation and angiogenesis 5, 10-12. Deletion of the TF cytoplasmic domain inhibits lipopolysaccharide-induced leukocyte recruitment and cytokine production 13, 14, suggesting its implication in Toll-like receptor 4 induced inflammation. The role of the TF cytoplasmic domain in angiogenesis is context dependent. In murine small intestine, the TF cytoplasmic domain promotes microbiota-induced angiogenesis via a PAR1-dependent pathway 15. Conversely, it inhibits PAR2-dependent angiogenesis during tumor expansion and in ischemic retinopathies 16. In the heart, PAR1 and PAR2 are implicated in cardiac injury and remodeling 17-20. In light of the aforementioned emerging evidence, we hypothesized that the TF cytoplasmic domain is involved in inflammatory or angiogenic processes in response to cardiac injury.

In this study, we investigated the involvement of the TF cytoplasmic domain in myocardial ischemic injury and post-infarct LV remodeling, and explored the underlying mechanisms.

## Materials and Methods

Detailed materials and methods, and all supporting data are available within the article and Data Supplement.

### Study Approval

All animal experiments were approved by the National University Singapore Institutional Animal Care and Use Committee (IACUC) and conformed to the guidelines on the care and use of animals for scientific purposes (NACLAR, Singapore, 2004) and the Guide for the Care and Use of Laboratory Animals published by the US National Institutes of Health (NIIH Publication, 8^th^ Edition, 2011).

### Animals and *In vivo* Experimental Procedures

All mice used for this study were of C57/BL6Jax background and maintained under a 12/12‐hour light‐dark cycle (lights on at 7 AM, lights off at 7 PM) at the Comparative Medicine Animal Vivarium at National University of Singapore. TF∆CT mice, which lack the 18 carboxy-terminal intracellular amino acids of TF were obtained from University of Leuven 10. The genotype of TF∆CT mice was confirmed by PCR (Figure S1). Wild-type (WT) C57/BL6 mice from Jackson laboratories were purchased from InVivos (Singapore). Male mice, unless stated otherwise, at 8 - 12 weeks old and 20 - 25 g were used for all experiments. Myocardial infarction (MI) was induced by permanent occlusion of the left anterior descending coronary artery (LAD) 21, 22. To ensure similar initial infarct size between WT and TF∆CT mice, myocardial area at risk was determined 24 hours after MI using Evans Blue and triphenyltetrazolium chloride staining 23. Mice that died prior to completion of the study protocol were used to determine mortality rates but excluded from other analyses. Chimeric mice were generated by bone marrow transplantation 24 and the chimerization was confirmed by flow cytometry analysis of peripheral blood (more than 95% leukocytes derived from donor bone marrow; Figure S2). The isotype controls and Fluorescence Minus One Controls (FMO) were used to define gates and interpret flow cytometry data. Data were analyzed using FlowJo (BD) software (v10.1). Osmotic minipump (Alzet 2004 - Rate: 0.25 μl/hr; Durect, Cupertino, CA) was implanted subcutaneously immediately before MI surgery as previously described 25 to infuse continuously the peptides (a PAR1 agonist peptide TFLLR-NH2, a PAR2 antagonist peptide FSLLRY-NH2, or a control peptide RLLFT-NH2) at 3 mg·kg^-1^·day^-1^ for 28 days in TF∆CT mice. Cardiac function was assessed with a high frequency ultrasound system Vevo® 2100 22. Speckle-tracking strain analysis (radial strain) was performed on long-axis B-mode images over three consecutive cardiac cycles with the VevoStrain software (Visualsonics) 23. All the data were analyzed with Vevo® 2100 software (version 1.7.0) by an experienced researcher who was blinded to the mouse genotypes and experimental procedures.

### Cell isolation, Cell Culture, Sorting and Staining

Isolation of cardiac resident cells including cardiomyocytes, fibroblasts, endothelial cells and resident immune cells, and culture of bone marrow (BM)-derived macrophages (BMDM) were described previously 22. Cardiac fibroblasts (Podoplanin^+^/CD45^-^/DAPI^-^), endothelial cells (CD31^+^/CD45^-^/DAPI^-^) and resident immune cells (CD45^+^/DAPI^-^) were isolated and sorted for immunofluorescence staining. Macrophage polarization toward M1 phenotype was induced by incubating BMDM with LPS and IFN-ɣ. Stained endothelial cells and cardiac resident immune cells were plated on pre-coated glass slides using Cytofuge 2 cytocentrifuge (Beckman Coulter). Apart from the isotype control antibodies, the FMO controls were used to define gates and interpret flow cytometry data. Data were analyzed using FlowJo (BD) software (v10.1). All the antibodies were listed in Table S1.

### mRNA and Protein Analysis

qPCR was performed with various primers listed in Table S2. Multiplex immunoassay was used to determine the concentrations of inflammatory cytokines and chemokines in the myocardium on a Bio-Plex 200 multiplex suspension array system (Bio-Rad). Matrix metalloproteinase zymography was used to examine the activation of matrix metalloproteinases (MMP) 2 and 9 in heart tissue 22. Western blot was used for analysis of protein expression levels of Rac1 and angiogenic factors. Blots were visualized with ChemiDoc Imager and quantified with ImageJ software.

### Histological analyses

To quantify the density and types of collagen in the infarct myocardium, paraffin-embedded heart tissue was stained with Picrosirius Red 24. Images were taken in bright field and circularly polarized filter and analyzed with Nikon AR element analysis software version 4.5.0 (Nikon Instrument Inc.). Protein expression (tissue factor, PAR1 and PAR2), infiltration of inflammatory cells (neutrophils, macrophages and T cells), cell activation and proliferation (fibroblasts and endothelial cells), and angiogenesis were analyzed by immunohistological staining. Cell specific markers used for histological staining include: Ly6G (BD Pharmingen, Heidelberg, Germany) for neutrophils, MAC3 (BD Pharmingen) for macrophages, CD3 (Dako, Glostrup, Denmark) for T cells, Ki67 (ThermoFisher) for proliferative cells, aSMA (Abcam) for activated myofibroblasts, isolectin B4 (IB4) for vascular endothelial cells, iNOS (inducible nitric oxide synthase enzyme; Abcam) for M1 macrophages, CD206 (Bio-Rad, Hercules, California, US) for M2 macrophages, CD68 (Bio-Rad) for pan macrophages.

### Statistical Analysis

Statistical analysis was performed using SPSS software (IBM® SPSS® Statistics version 22.0). Gaussian distribution was assessed, and the appropriate Student's t-test or Mann-Whitney U test was used to compare two groups, with one-way ANOVA or two-way repeated measures ANOVA followed by Bonferroni *post hoc* testing of multi-group comparisons. Kaplan-Meier analysis with log rank testing was used for inter-group comparisons of mortality. Sample sizes are indicated in the figure legends. Heatmaps for cytokines and chemokines were generated by Prism 8.0 software (GraphPad). Data are presented as mean ± SEM, unless stated otherwise. *P* < 0.05 is considered statistically significant.

## Results

### Myocardial expression of TF decreases in the infarcted myocardium

As previously reported 7, 9, TF is highly expressed in LV myocardium (Figure S3). Following MI, TF expression in the myocardium, as demonstrated by immunofluorescent staining, decreased (Figure S3A). Accordingly, mRNA expression of TF decreased in the infarcted myocardium (Figure S3B). Compared with sham, myocardial expression of TF significantly declined at 3 days post-MI with partial recovery at 7 days, in both WT and TFΔCT mice. The 7-day recovery of TF expression was less in TFΔCT mice compared with WT mice. The change of TF expression in the infarcted myocardium over time was likely due to dynamic infiltration of various TF-expressing inflammatory cells, including neutrophils, T cells and macrophages (Figure S4).

### Lack of the TF cytoplasmic domain promotes post-MI survival, alleviates post-MI cardiac dysfunction and adverse LV remodeling

**Figure 1:**
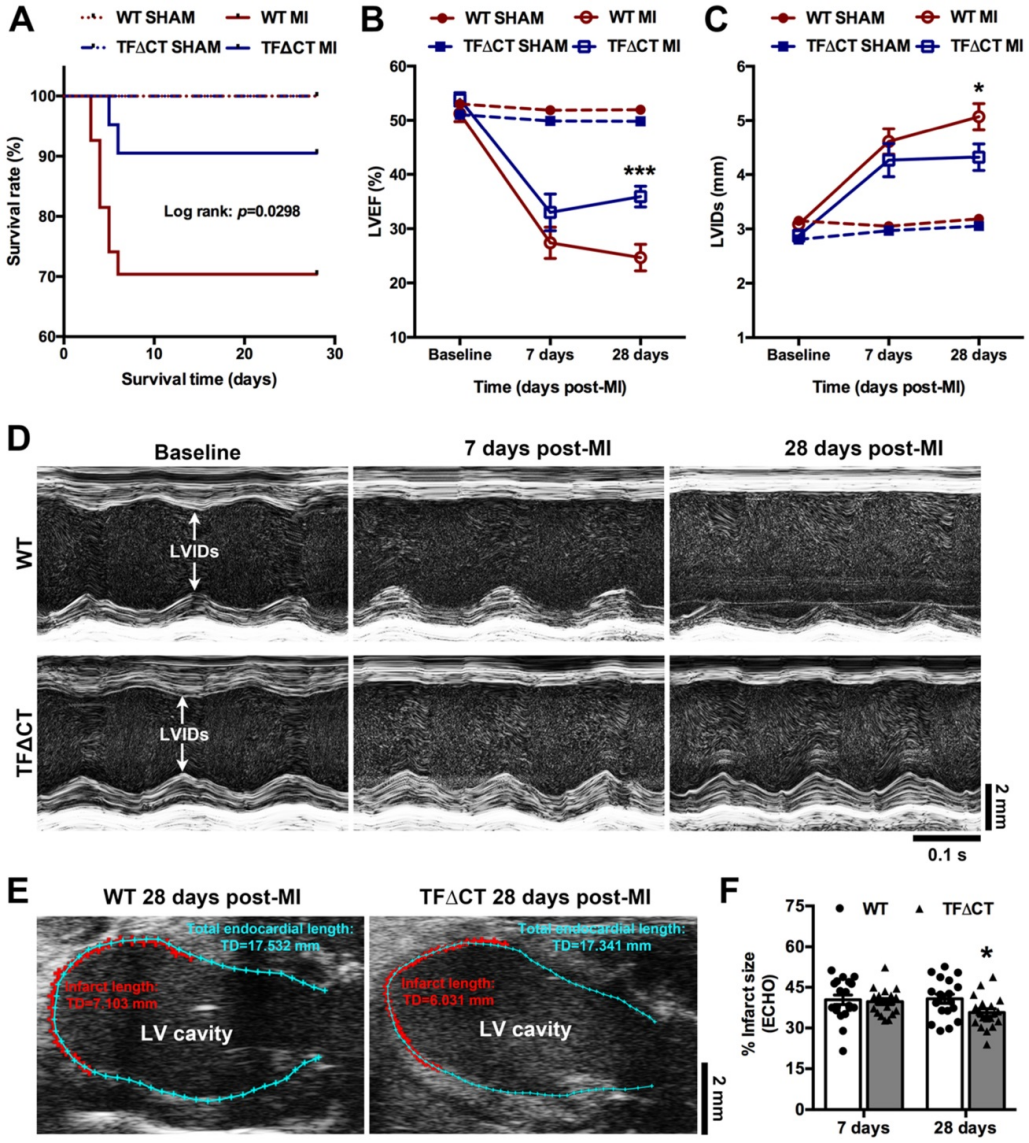
** Lack of the TF cytoplasmic domain promotes survival and protects cardiac function after MI.** (**A**) The Kaplan-Meier survival curves. Log-rank test; n = 27 for WT mice and n = 21 for TF∆CT mice subjected to MI, n = 10 for WT and n = 9 for TF∆CT sham groups. (**B** and** C**) LVEF and LVIDs determined by echocardiography. *P < 0.05, ***p < 0.001 compared with WT mice by two-way ANOVA with Bonferroni *post hoc* test; n = 19 for WT mice and n = 19 for TF∆CT mice surviving MI, n = 10 for WT and n = 9 for TF∆CT sham groups. (**D**) Representative echocardiograms (M-mode tracing) illustrating cardiac changes after MI in WT versus TF∆CT mice. (**E** and **F**) Post-MI infarct size was estimated by measuring the length of myocardial infarct (in RED) and total length of LV endocardium (in CYAN) at the middle plane of the long-axis LV echocardiogram as indicated. Infarct size (%) = (length of infarct / length of LV endocardium) x 100. *P < 0.05, compared with WT, two-way ANOVA with Bonferroni *post hoc* test for multiple comparison. LVEF, left ventricular ejection fraction; LVIDs, internal diameter at end of systole.

TFΔCT mice are viable and develop normally after birth, as previously described 10. At baseline, TFΔCT and WT mice did not differ in any of the cardiac parameters measured (Table 1). TFΔCT mice, however, showed a better post-MI survival than WT mice (Figure 1A). Five mice per genotype were randomly chosen at 24 hours post-MI for measuring area at risk. Histological staining demonstrated that the initial myocardial injury induced by MI did not differ between WT and TFΔCT mice (Figure S5A-B), indicating that the difference in post-MI survival was solely attributable to mouse genotypes. The results were confirmed by plasma levels of troponin I (Figure S5C).

Among surviving mice, cardiac parameters were determined by echocardiography (Table 1 and Figure 1B-D). Decline in cardiac systolic function after MI, determined by LV ejection fraction and fraction shortening, did not differ between WT and TFΔCT mice at 7 days. At 28 days, TFΔCT mice showed smaller LV volumes at end systole and end diastole resulting in bigger ejection fraction. Similarly, LV internal diameters at end systole and end diastole were larger in WT mice compared with TFΔCT mice at 28 days (Table 1). The infarct scar, as estimated by echocardiography (Figure 1E), was comparable between WT and TFΔCT mice within the first week, however, it was significantly larger in WT mice at 4 weeks after MI, likely due to more severe dilation of LV in WT mice (Figure 1F).

### Late improvement of myocardial contractility within infarct-and-border region contributes to attenuated LV remodeling in infarcted TF∆CT mice

**Figure 2:**
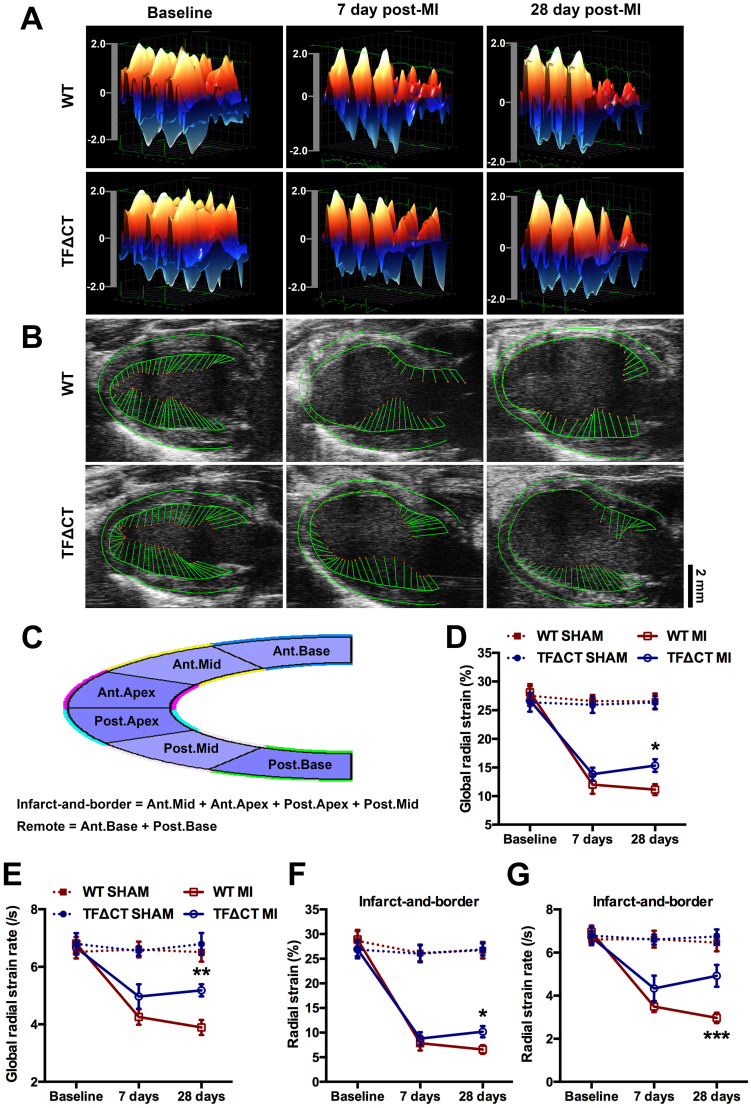
** Lack of the TF cytoplasmic domain preserves cardiac contractility and attenuates myocardium deformation after MI.** (**A**) Three-dimensional (3D) regional wall velocity diagrams of LV endocardial strain illustrating contraction (red-orange) and relaxation (blue) of three consecutive cardiac cycles. (**B**) Vector diagrams illustrating the direction and magnitude of endocardial contraction at mid-systole. (**C**) A schematic diagram illustrating global and regional strain analysis. (**D**-**G**) Global and regional radial strain determined by speckle-tracking strain analysis. *P < 0.05, **p < 0.01 and ***p < 0.001 compared with WT mice by two-way ANOVA with Bonferroni *post hoc* test; n = 19 for WT mice and n = 19 for TF∆CT mice subjected to MI, n = 10 for WT and n = 9 for TF∆CT sham groups. Ant.Base, anterior base; Post.Base, posterior base, Ant.Mid, anterior middle; Post.Mid, posterior middle; Ant.Apex, anterior apex; Post.Apex, posterior apex.

To further examine post-MI LV remodeling, we performed strain analysis using high-frequency speckle tracking echocardiography that depicts myocardial contractility and geographical changes (Figure 2).

Three-dimensional (3D) wall velocity diagrams (Figure 2A) showed that all hearts exhibited uniform contraction and relaxation throughout the LV endocardium at baseline. Following MI, wall velocity of the infarct-related anterior wall fell similarly in WT and TFΔCT mice at 7 days post-MI but increased to be significantly higher in TFΔCT mice by 28 days post-MI. In addition, vector diagrams and videos showed higher vector activities in the infarct border region of TFΔCT hearts compared with WT hearts at 28 days post-MI (Figure 2B and Videos I-IV).

Geographical changes in myocardium were analyzed by radial strain and strain rate calculated based on the semi-automated segmentation (Figure 2C). After MI, global radial strain and strain rate declined in WT and TFΔCT mice to a similar extent at 7 days (Figure 2D,E). At 28 days, however, TFΔCT mice showed higher strain and strain rate compared with WT mice. Furthermore, regional strain analysis revealed that TFΔCT mice, compared with WT mice, had higher radial strain and strain rate in the infarct-and-border region with no significant difference in radial strain or strain rate detected in the remote region of LV (Figure 2F,G and Figure S6).

### Absence of the TF cytoplasmic domain in either cardiac resident cells or infiltrating cells alleviates post-infarct LV remodeling

TF was expressed in most cardiac resident cells (fibroblasts, cardiomyocytes and macrophages) and bone marrow (BM)-derived macrophages (BMDM; Figures S4 and S7). To identify the cellular source of the TF cytoplasmic domain contributing to post-MI LV remodeling, we generated BM chimeras as previously described 24. All chimeric mice survived MI to 28 days: WT mice receiving TFΔCT BM (referred as WT/TFΔCT BM), TFΔCT/WT BM, WT/WT BM and TFΔCT/TFΔCT BM (Table S3). Among the 4 chimeras, WT/WT BM mice showed lowest ejection fraction and fraction shortening compared with the other 3 chimeras. In addition, WT/WT BM mice had significantly larger end systolic volume than WT/TFΔCT BM and TFΔCT/TFΔCT BM mice. No clear difference in cardiac function parameters was observed among TFΔCT/WT BM, WT/WT BM and TFΔCT/TFΔCT BM groups.

### Attenuated LV remodeling in TF∆CT mice is associated with favorable modulation of extracellular matrix

**Figure 3:**
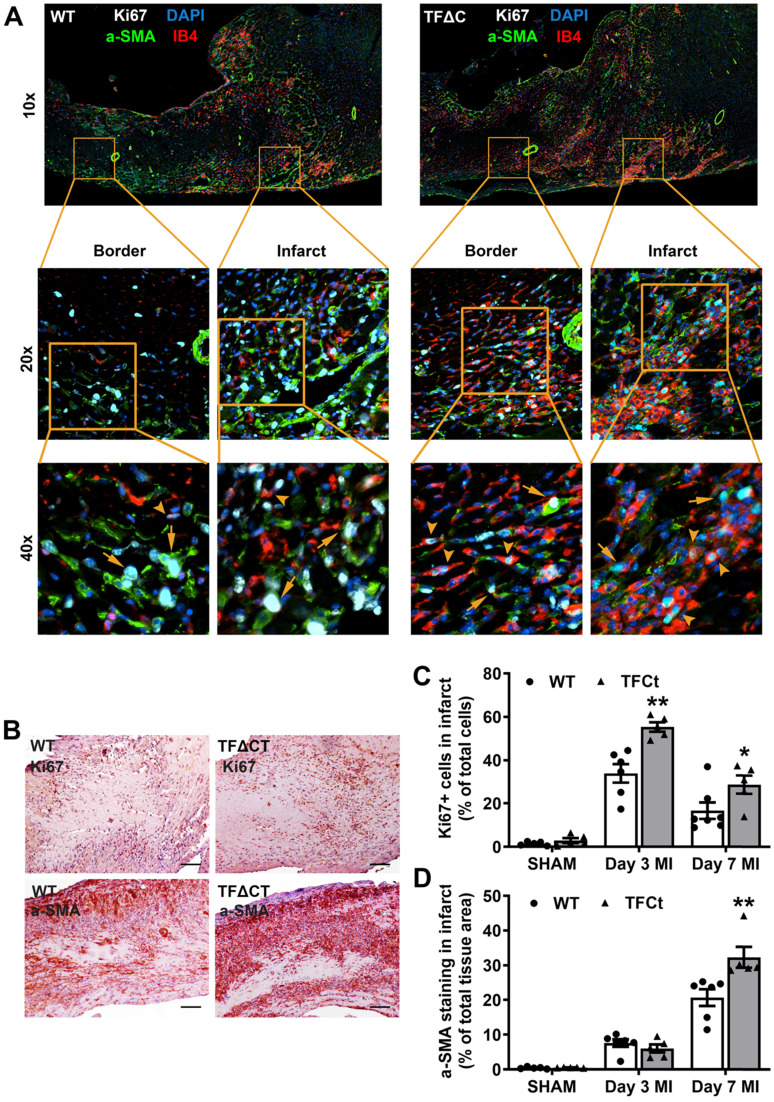
** Lack of the TF cytoplasmic domain enhances proliferation of myofibroblasts and endothelial cells after MI.** (**A**) Representative heart setions (3 days post-MI) showing proliferation of Acellular matrix areas and expression of extracellular matrix proteins. Fibroblasts were stained by a-SMA and endothelial cells by IB4. Arrows indicate Ki67 positive fibroblasts and arrowheads indicate Ki67 positive endothelial cells. Images were taken by at 10x, 20x or 40x by Nikon Eclipse Ti-E inverted microscope. (**B**) Staining of a proliferative marker Ki67 at 3 days post-MI and myofibroblast marker α-SMA at 7 days post-MI. Scale bars represent 100 µM. (**C** and **D**) Quantification of Ki67 and α-SMA staining in the infarcted hearts. N = 5-6 per genotype per time-point. Mann-Whitney U test, *p < 0.05, **p < 0.01 compared with WT mice.

**Figure 4:**
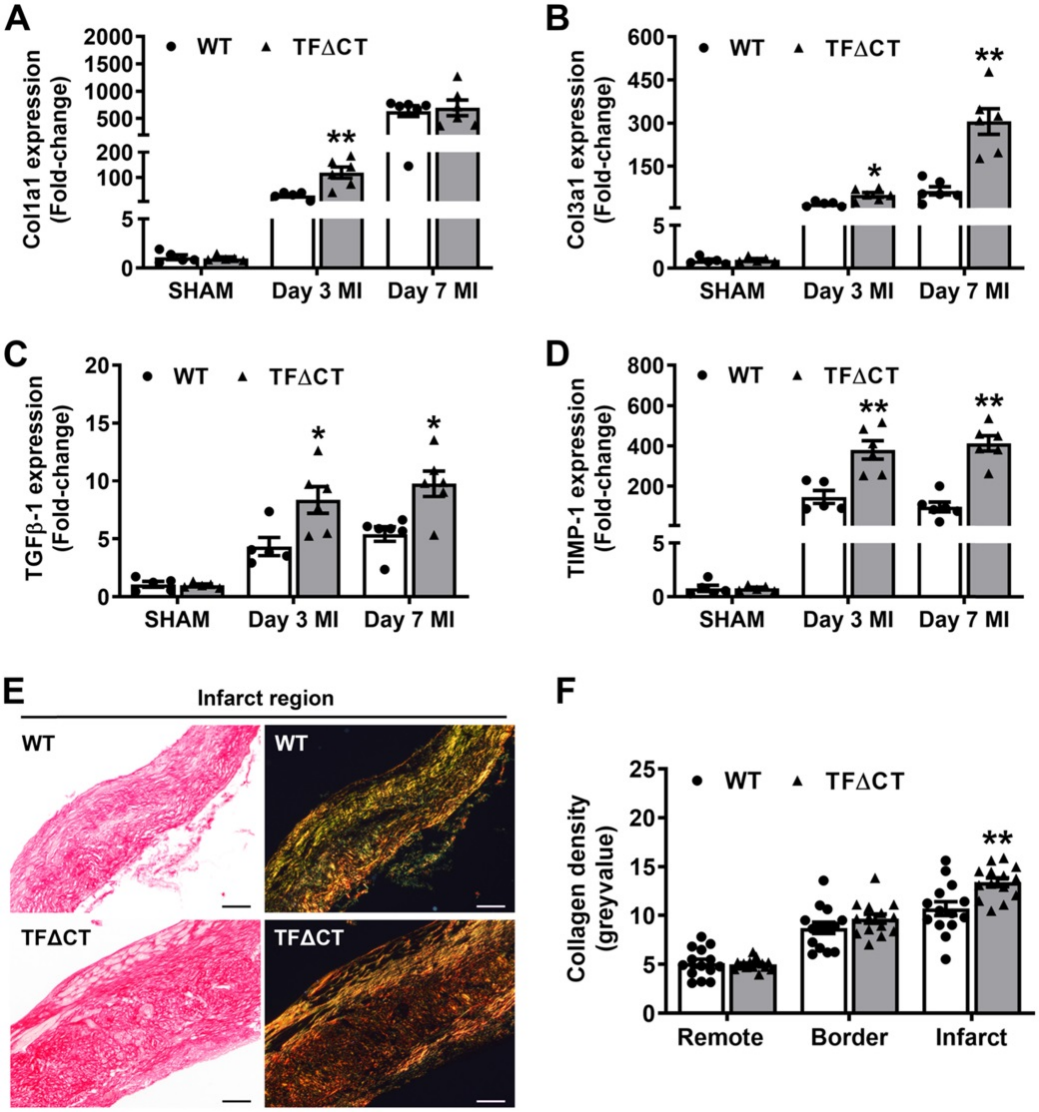
** Lack of the TF cytoplasmic domain promotes post-MI cardiac extracellular matrix synthesis and scar formation.** (**A**-**D**) Relative mRNA expression of Col1a1, Col3a1, TGFβ-1 and TIMP-1 was determined in the infarcted myocardium. Gene expression was normalized to sham-operated mice of its own genotype. N = 5-6 per genotype per time-point. (**E**) Representative heart sections (28 days post-MI) stained with Picrosirius Red, imaged under white light or polarized light. Scale bars represent 100 µM. (**F**) Quantification of collagen contents in the infarct 28 days post-MI (n = 14 per group). Mann-Whitney U test, *p < 0.05, **p < 0.01 compared with WT mice.

Having demonstrated the attenuated LV remodeling in TFΔCT mice, we examined cardiac ECM which is essential for post-MI cardiac repair and LV remodeling 3. Compared with WT hearts, TFΔCT hearts exhibited a larger area of acellular matrix (granular tissue formed following cardiomyocyte death) at 7 days post-MI (Figure S8), reflecting a better preservation of ECM in infarcted TFΔCT hearts. Cell proliferation, as indicated by Ki67 staining, was significantly enhanced in infarcted TFΔCT hearts with fibroblasts and endothelial cells being the major cell populations (Figure 3A-D). Importantly, the increased staining of α-smooth muscle actin (α-SMA) suggests that more cardiac fibroblasts were activated and differentiated into reparative myofibroblasts in infarcted TFΔCT hearts (Figure 3D). Accordingly, infarcted TFΔCT hearts expressed higher mRNA levels of matricellular proteins including osteopontin and thrombospondin 1 (Figure S8). Collagens, the main components of ECM secreted by (myo)fibroblasts, increased in response to MI as previously reported 22 (Figure 4A,B). In TFΔCT hearts, Col1a1 and Col3a1 mRNA levels were higher compared with WT with expression of Col3a1 5-fold higher than that in WT hearts at 7 days post-MI. Infarcted TFΔCT hearts showed also significantly higher mRNA expression of transforming growth factor β1 (TGF-β1), a cytokine promoting collagen synthesis 26, at 3 and 7 days post-MI (Figure 4C).

Besides matrix production, we also examined ECM degradation pathways. Both mRNA levels and protease activities of MMPs 2 and 9, the main proteases of cardiac collagens 27, increased after infarction but did not differ between WT and TFΔCT hearts (Figure S9). However, mRNA expression of tissue inhibitor of metalloproteinase 1 (TIMP-1), an endogenous inhibitor of MMPs 28, increased 100 folds after MI to be ~2-4 times higher in infarcted TFΔCT hearts (Figure 4D). Further analysis showed that TIMP-1 was mainly produced by cardiac (myo)fibroblasts (Figure S10).

The higher expression of collagens and TIMP-1 in TFΔCT hearts suggest that the balance between collagen production and degradation has moved towards more collagen and collagen fibers in the heart after infarction. Therefore, we analyzed the maturity of collagen fibers based on their optical properties: the immature and fibrillar fibers appear green in color, whereas mature and thick fibers appear yellow-orange and the most mature ones appear red in color 29, 30. Indeed, cardiac collagens in infarcted TFΔCT hearts resulted in a more mature scar replacing dead myocardium, as shown by denser collagen fibers within the infarct at 28 days after MI (Figure 4E,F). Collagen deposition in the remote and infarct border regions, however, did not differ between WT and TFΔCT hearts (Figure 4F and Figure S11).

### Lack of the TF cytoplasmic domain attenuates post-MI inflammatory response

**Figure 5:**
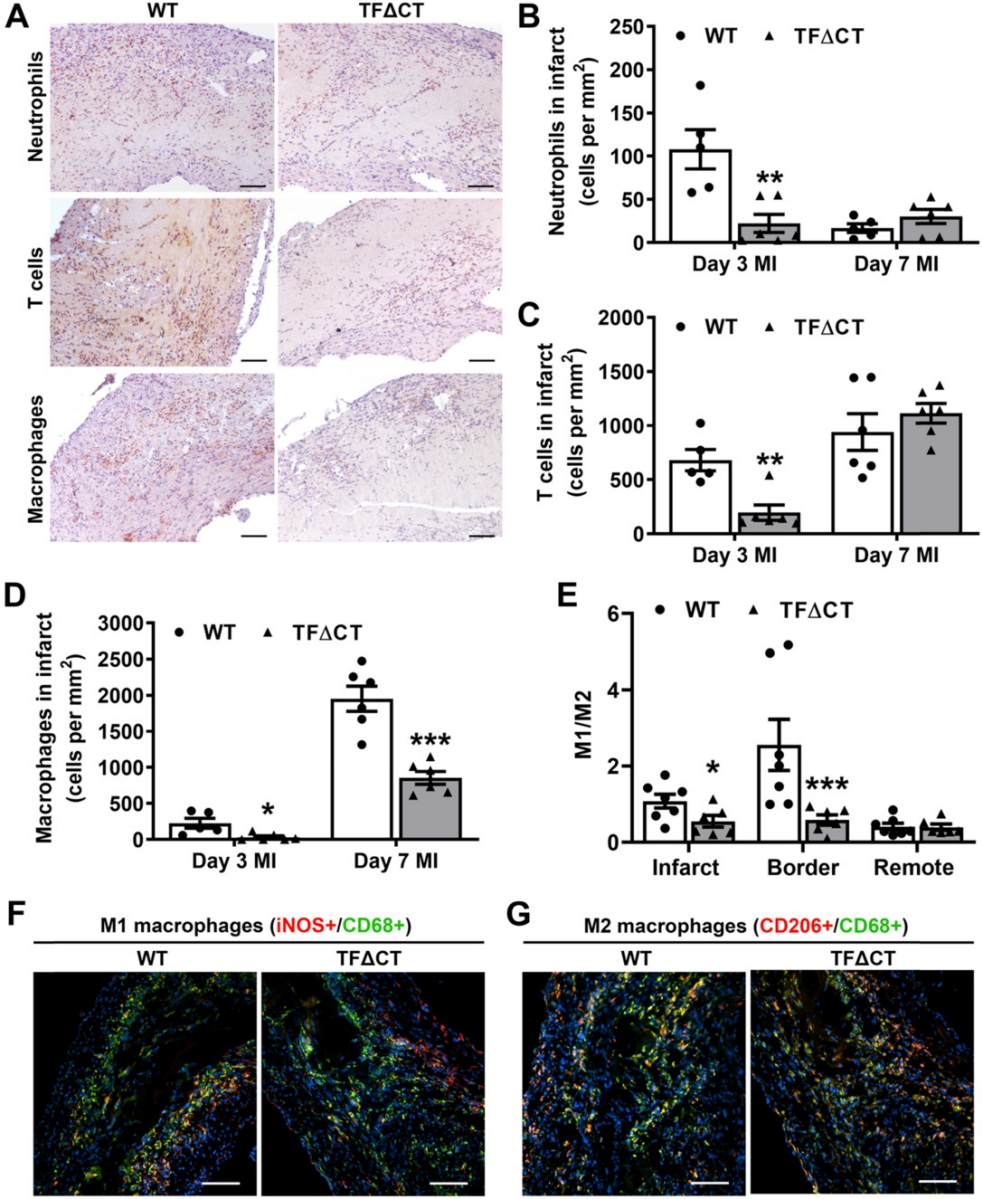
** Lack of the TF cytoplasmic domain attenuates immune cell influx in response to MI.** (**A**) Representative images of infarcted heart sections (3 days post-MI) stained for neutrophils, T cells and macrophages. Cell specific markers: Ly6G for neutrophils, CD3 for T-cells and MAC3 for macrophages. (**B**, **C** and **D**) Quantification of infiltrated cells in the infarct region. (**E**) Ratios of M1 to M2 macrophages in infarcted hearts (7 days post-MI). (**F** and **G**) Representative images of infarcted heart sections (7 days post-MI) stained for M1 (iNOS^+^) and M2 (CD206^+^) macrophages. N = 5 - 7 per genotype per time-point. Mann-Whitney U test, *p < 0.05, **p < 0.01, ***p < 0.001 compared with WT mice. Scale bars represent 100 µM.

Given the critical role of inflammation in myocardial injury and LV remodeling following MI, we determined immune cell infiltration and cytokine production in the heart. Compared with WT hearts, TFΔCT hearts had fewer neutrophils and T cells infiltrated into the infarct and border regions at 3 days post-MI (Figure 5A-C and Figures S12-S13). At 7 days, WT and TFΔCT hearts had similar amounts of infiltrating neutrophils and T cells in the infarct region, however, TFΔCT hearts hosted higher amounts of neutrophils and T cells in the border region (Figure 5 and Figures S12-S13). Concomitantly, TFΔCT hearts showed fewer infiltrating macrophages in both infarct and border regions at 3- and 7-days post-MI compared with WT hearts (Figure 5D and Figures S12-S13). Immunofluorescence staining revealed that WT hearts, compared to TFΔCT hearts, exhibited higher percentage of pro-inflammatory M1 macrophages (iNOS^+^/CD68^+^) at both infarct and border regions, but lower percentage of reparative M2 macrophages (CD206^+^/CD68^+^), resulting in higher ratios of M1/M2 macrophages in WT hearts (Figure 5E-G, Figure S14, and Figure S15A). Furthermore, our *in vitro* experiments demonstrated that polarization of primary macrophages (BMDM) derived from TFΔCT mice towards M1 phenotype (induced by LPS and IFN-ɣ) was remarkably inhibited compared with macrophages derived from WT mice (Figure S15B).

**Figure 6:**
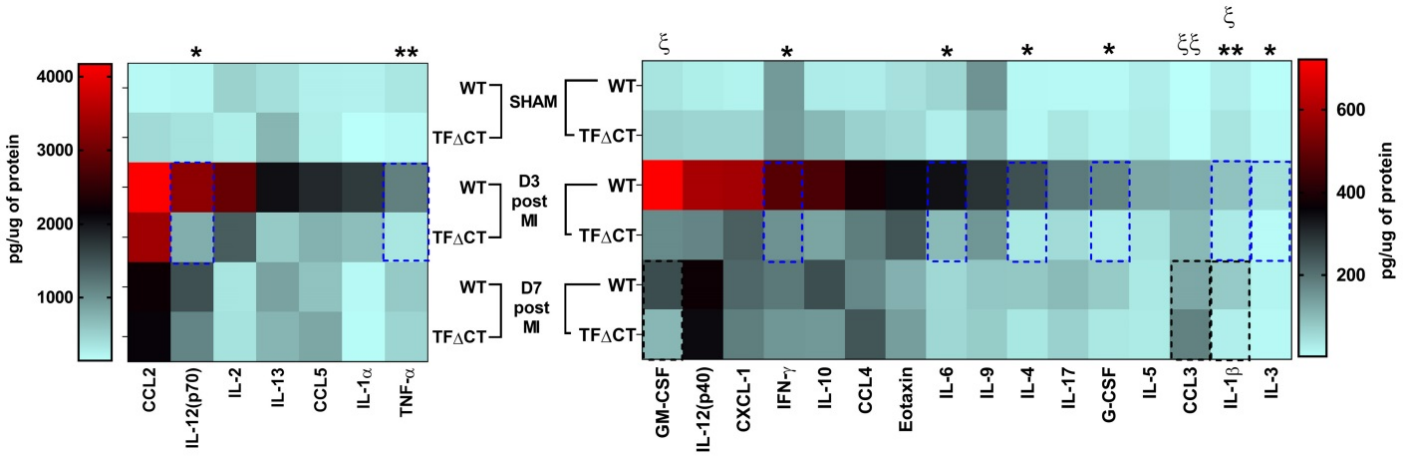
** Lack of the TF cytoplasmic domain decreases cytokine and chemokine production post-MI.** Protein concentrations of cytokines and chemokines in lysates of infarcted myocardium were quantified by multiplex assay at different time-points post-MI. N = 5 - 6 per genotype per time-point; Kruskal-Wallis followed by Dunn *post hoc* test., *p < 0.05, **p < 0.01 at 3 days post-MI; **^ξ^***p* < 0.05, **^ξ ξ^***p* < 0.01 at 7 days post-MI compared to WT mice.

To further characterize the inflammatory response, we measured the protein levels of cytokines and chemokines in the infarcted myocardium with a multiplex assay (Figure 6 and Table S4). At 3 days post-MI, protein levels of most detected cytokines and chemokines were significantly lower in infarcted TFΔCT hearts than in WT hearts. At 7 days, lower levels of IL-1β and GM-CSF, and higher levels of chemokine CCL3, were detected in TFΔCT hearts whilst protein levels of other cytokines and chemokines were similar between WT and TFΔCT hearts. In the remote myocardium, levels of cytokines or chemokines did not differ between WT and TFΔCT hearts at any time-point.

To confirm the inflammatory role of the TF cytoplasmic tail at cellular level, we determined the inflammatory response of BMDM *in vitro*. Upon LPS stimulation, the upregulation of pro-inflammatory cytokines, CCL2, IL-6, and IL-1β, was drastically inhibited in BMDM derived from TF∆CT mice (Figure S16).

### Lack of the TF cytoplasmic domain accelerates angiogenesis following MI

**Figure 7:**
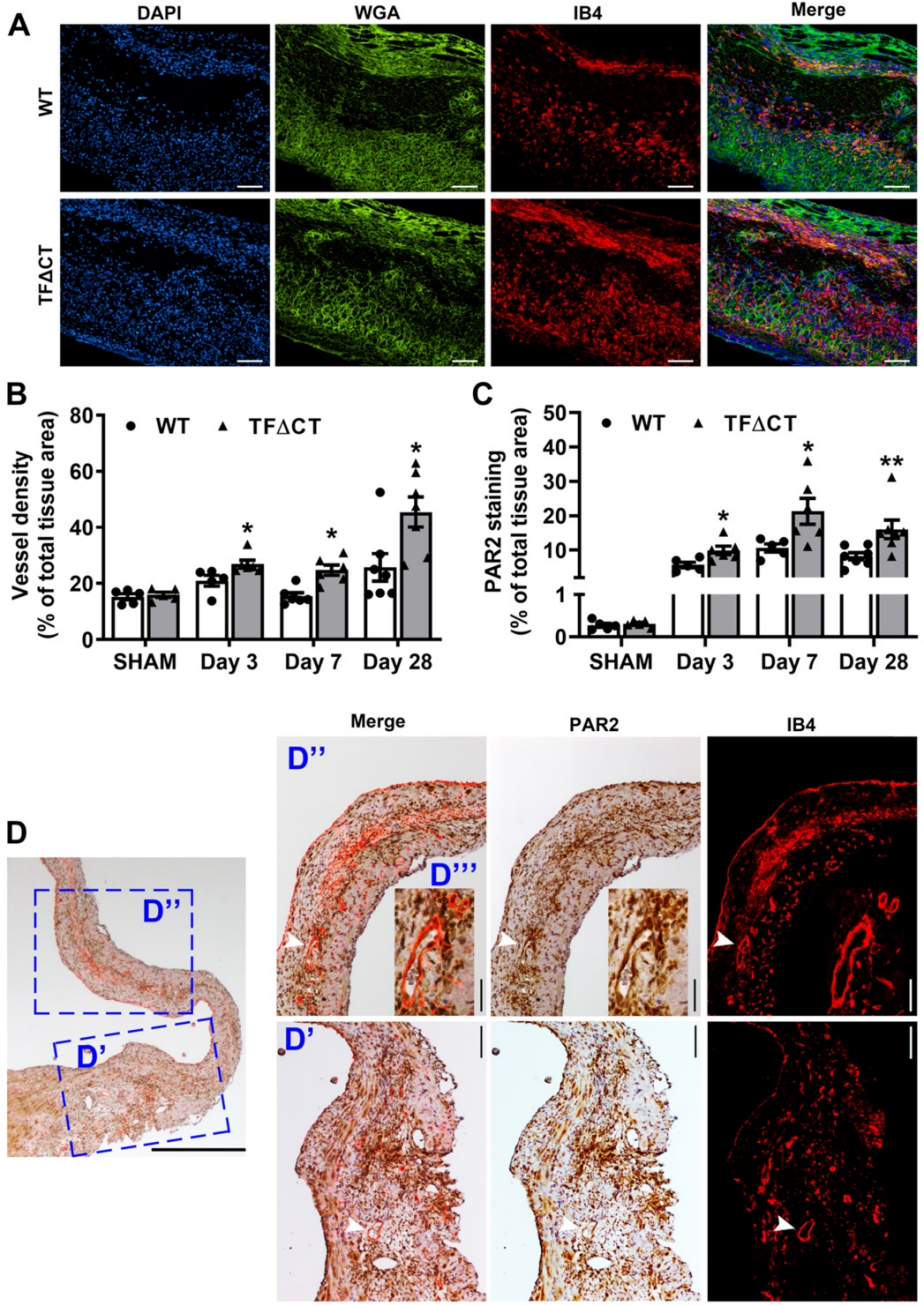
** Lack of the TF cytoplasmic domain promotes angiogenesis in infarcted myocardium.** (**A**) Representative images of infarcted heart sections stained with DAPI (blue), WGA (green) and IB4 (red) at 7 days post-MI. (**B** and **C**) Quantification of blood vessel density and PAR2 staining in the infarct-and-border region. N = 5 - 7 per genotype per time-point; Mann-Whitney U test, *p < 0.05, **p < 0.01 compared with WT mice. (**D**) A representative heart section of a TF∆CT mouse at 28 days post-MI demonstrating co-localization of PAR2 with myocardial capillaries (stained with IB4). Panels (**D'**, **D'')** show close-up of infarct border and infarct regions, respectively. An inset (**D'''**) shows close-up of myocardial capillaries in the infarct region. White arrowheads indicate co-localization of PAR2 with capillaries. Scale bars: 100 µM.

Angiogenesis is a critical adaptive mechanism for restoration of myocardial perfusion after MI 4, we therefore examined if the TF cytoplasmic domain contributes to MI-induced angiogenesis. At baseline, myocardial vessel density, as demonstrated by isolectin-B4 staining, was similar between WT and TFΔCT mice (Figure S17). Compared with WT hearts, the vessel density was significantly higher in the infarct-and-border region of TFΔCT hearts (Figure 7A,B), suggesting a greater neovascularization in absence of the TF cytoplasmic domain. This enhanced angiogenesis in TFΔCT hearts likely resulted from a higher proliferation of endothelial cells in response to MI (Figure 3A). The unique clustering of newly formed capillaries in infarcted TFΔCT hearts may account for the improved regional radial strain and strain rate within the infarct-and-border region (Figure 2F,G).

### The TF cytoplasmic domain may regulate post-infarct myocardial inflammation and angiogenesis via PAR1 and PAR2 associated pathways

To explore the TF cytoplasmic domain downstream pathways potentially involved in post-infarct myocardial injury, we examined cardiac mRNA expression of PAR1, PAR2 and actin-binding protein-280 (ABP-280)/Filamin A following MI. mRNA levels of PAR1 and PAR2 (Figure S18) increased in both WT and TFΔCT hearts at 3- and 7-days post-MI compared with sham. At 7 days, PAR1 mRNA levels were lower in TFΔCT hearts (compared to sham, an 18-fold increase for WT versus a 10-fold increase for TFΔCT). In contrast, PAR2 mRNA levels were higher in TFΔCT hearts at 7 days post-MI. ABP-280/Filamin A that mediates the TF cytoplasmic domain signaling in tumor cell metastasis and vascular remodeling 31 increased in response to MI but to a similar extent in WT and TFΔCT mice (Figure S19).

The concurrent reduction of inflammation (Figure 5 and Figure 6) and PAR1 expression (mRNA levels in Figure S18 and protein levels in Figure S20) in infarcted TFΔCT hearts compared with infarcted WT hearts prompted us to examine the protein levels of Rac1, a small GTPase involved in the TF cytoplasmic domain-PAR1 axis mediated inflammatory response 32, 33. As shown by Western blot, Rac1 expression increased following MI but to a lesser extent in infarcted TFΔCT hearts (Figure S21).

The TF cytoplasmic domain has been reported to regulate angiogenesis via PAR2 signaling 16. As demonstrated by immunofluorescence staining, PAR2 was diffusely distributed in the myocardium at baseline (Figure S17B). Following MI, PAR2 protein levels increased in both WT and TFΔCT hearts (Figure 7C). Compared with WT hearts, TFΔCT hearts displayed a higher abundance of PAR2 in the infarct-and-border region. Furthermore, PAR2 co-localized with myocardial neovessels (Figure 7D).

**Figure 8:**
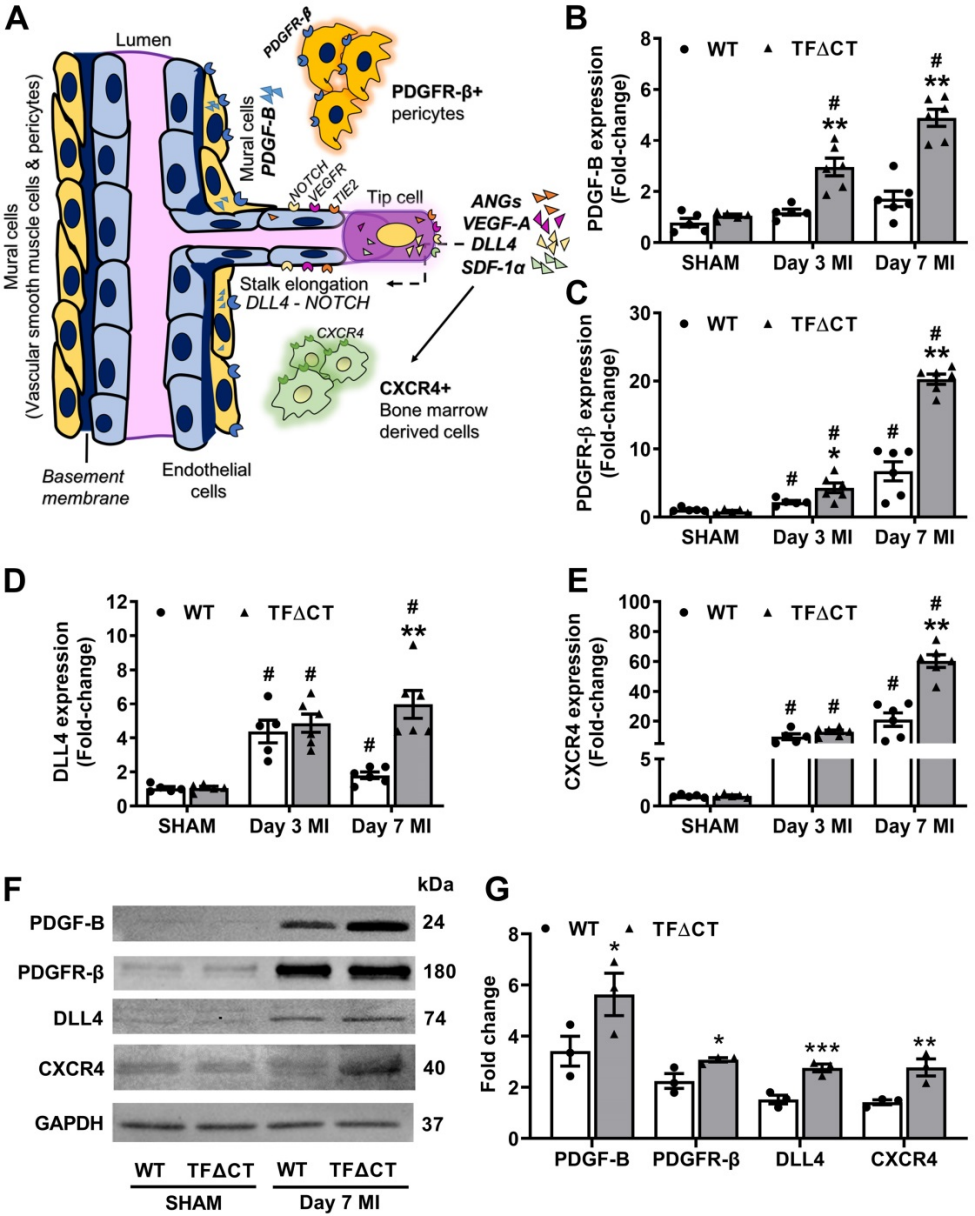
** The TF cytoplasmic domain regulates angiogenic signaling pathways in the infarcted heart.** (**A**) A schematic diagram illustrating key angiogenic signaling molecules examined in the infarcted heart. (**B**-**E**) mRNA expression levels of PDGF-B and its receptor PDGFR-β, NOTCH ligand DLL4, SDF-1α receptor CXCR4, in the infarcted myocardium. Relative mRNA expression was normalized to sham-operated mice of its own genotype. N = 5 - 6 per genotype per time-point. (**F**) Western blot analysis of PDGF-B, PDGFR-β, DLL4 and CXCR4 protein levels in the infarcted myocardium from mice with SHAM surgery or 7 days post MI. (G) Quantification of protein levels analyzed by Western blot in panel **F**. N = 3 per genotype of mice. Protein levels were normalized to SHAM and presented as fold changes. Mann-Whitney U test, *p < 0.05, **p < 0.01 compared with WT mice; #*p* < 0.05 compared with sham. PDGF-B, platelet derived growth factor subunit B; PDGFR-β, PDGF-B receptor; DLL4, Delta Like Canonical Notch Ligand 4; SDF-1α, stromal cell-derived factor 1; CXCR4, C-X-C chemokine receptor type 4.

To identify the angiogenic pathways involved in the TF cytoplasmic domain-PAR2 signaling in the heart, we examined the expression of several key angiogenic factors (Figure 8A). We found that the mRNA expression of vascular endothelial growth factor A (VEGF-A), angiopoietins 1 and 2, platelet derived growth factor subunit B (PDGF-B) and its receptor platelet-derived growth factor receptor beta (PDGFR-β), NOTCH ligand Delta like canonical Notch ligand 4 (DLL4), stromal cell-derived factor 1 alpha (SDF-1α) and its receptor C-X-C chemokine receptor type 4 (CXCR4), increased in the infarcted myocardium (Figure S22 and Figure 8B-E). Among these angiogenic factors, mRNA expression of VEGF-A showed a transient increase at 3 days post-MI with no difference between WT and TFΔCT hearts; whereas mRNA expression of angiopoietin 1 transiently decreased at 3 days and expression of angiopoietin 2 persistently increased at 3 and 7 days in response to MI, with no difference between WT and TFΔCT hearts (Figure S22B-D). Interestingly, the perivascular cell recruiting factors (PDGF-B/PDGFR-β axis) showed higher mRNA levels in TFΔCT hearts than WT hearts at both 3 and 7 days post-MI, and the vascular sprouting regulators (DLL4, SDF-1α and CXCR4) showed higher mRNA levels in TFΔCT hearts than WT hearts at 7 days post-MI (Figure 8B-E and Figure S22E). Higher expression of the perivascular cell recruiting factors and the vascular sprouting regulators in TFΔCT hearts than WT hearts at 7 days post-MI was further confirmed by Western blot analysis (Figure 8F,G and Figure S22F).

To identify the cellular source of these angiogenic factors, we isolated different cardiac resident cells, including cardiomyocytes, fibroblasts and endothelial cells, by FACS sorting, and determined the expression of angiogenic factors in those cardiac resident cells and BMDM (mimicking infiltrating macrophages). qPCR analysis showed that all the five angiogenic factors (DLL4, SDF-1α, PDGF-B, PDGFR-β and CXCR4) affected by the TF cytoplasmic domain were predominantly expressed in cardiac endothelial cells, with only CXCR4 also expressed in BMDM (Figure S23).

### Synergistic regulation of PAR1 and PAR2 activities abolishes cardiac protection in TF∆CT mice

**Figure 9:**
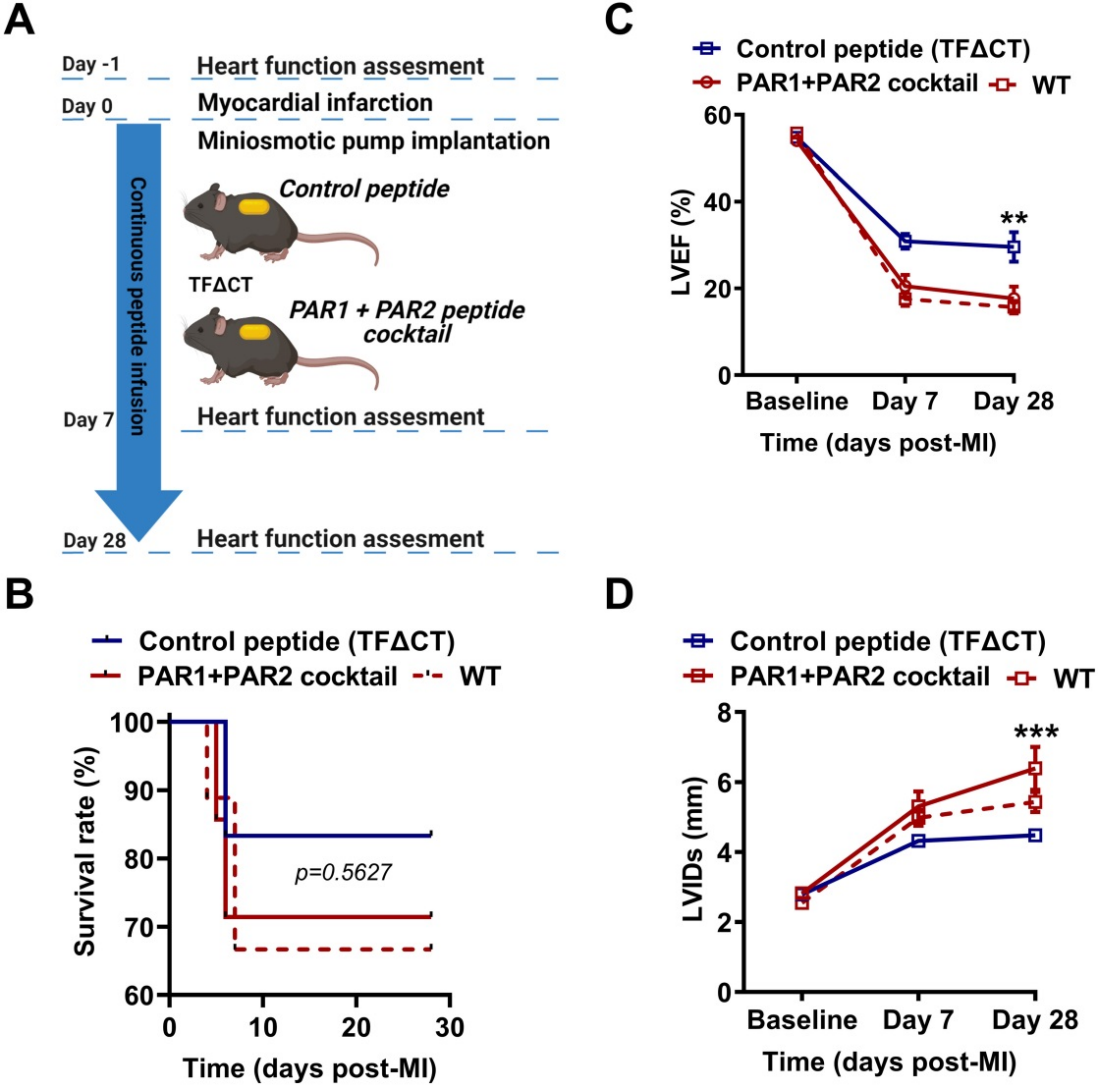
** Synergistic activation of PAR1 and inhibition of PAR2 activity abolish cardioprotection in TFΔCT mice. (A)** Experimental outline. TFΔCT mice, immediately prior to MI, were implanted with osmotic minipump supplemented with either the cocktail of PAR1 agonist and PAR2 antagonist peptides or the control peptide. Cardiac function was monitored by echocardiography. **(B)** The Kaplan-Meier survival curves. Log-rank test; n = 6 for TFΔCT mice infused with the control peptide, and n = 7 for TFΔCT mice infused with the cocktail of PAR1 agonist and PAR2 antagonist peptides, and n = 9 for WT mice serving as a control group. **(C, D)** LVEF and LVIDs determined by echocardiography for survived mice. **P < 0.01, ***p < 0.001 compared with TFΔCT mice infused with the control peptide by two-way ANOVA with Bonferroni *post hoc* test; n = 5 for both control peptide group and treatment group of TFΔCT mice, and n = 6 for WT mice. Of note, in panel **D**, the difference between WT mice and TFΔCT mice infused with the cocktail of PAR1 agonist and PAR2 antagonist peptides is not significant, but the difference between WT mice and TFΔCT mice infused with the control peptide was highly significant (*p* < 0.01).

To confirm the direct and synergistic contribution of PAR1 and PAR2 to the downstream signaling of the TF cytoplasmic domain in MI, we infused TFΔCT mice constantly for 28 days with a cocktail of PAR1-specific antagonist peptide (TFLLR-NH2) and a PAR2-specific agonist peptide (FSLLRY-NH2) by osmotic minipumps (Figure 9A). During 28 days of post-MI follow-up, five out of six TFΔCT mice receiving the control peptide survived (83%) while five out of seven TFΔCT mice receiving the cocktail of PAR1 agonist and PAR2 antagonist survived (71%). The survival rates between control and treatment groups did not significantly differ due to the small sample size (Figure 9B). However, compared with control peptide group, TFΔCT mice receiving the cocktail of PAR1 agonist and PAR2 antagonist post-MI displayed markedly lower LVEF and larger LVIDs (Figure 9C,D). Strikingly, TFΔCT mice receiving the cocktail of PAR1 agonist and PAR2 antagonist resembled WT mouse phenotype, including post-MI survival rate and cardiac dysfunction (Figure 9B-D). These results indicate that the cardioprotective effects observed in TFΔCT mice were largely attributable to decreased PAR1 activity and increased PAR2 activity induced by the lack of TF cytoplasmic domain.

## Discussion

The TF cytoplasmic domain has been implicated in inflammation and angiogenesis under pathological conditions such as infection, cancer and diabetes 13-15, 34. Inflammation and angiogenesis are two important components of wound healing and fibrosis underpinning post-infarct LV remodeling. In this study, we demonstrate that the TF cytoplasmic domain exacerbates post-infarct cardiac injury and adverse LV remodeling via differential regulation of inflammation and angiogenesis through PAR1 and PAR2 signaling, respectively. Genetic deletion of the TF cytoplasmic domain results in improved post-MI survival, less severe LV remodeling and less cardiac dysfunction. Based on our findings, we now propose a working model in which the TF cytoplasmic domain promotes PAR1-dependent inflammation whilst inhibiting PAR2-dependent angiogenesis in the ischemic heart, thereby contributing to adverse post-infarct LV remodeling.

We firstly confirmed previous reports 7, 9 that TF is highly expressed in murine LV myocardium and located at the intercalated discs. In this study, we found that TF expression decreased in the infarcted myocardium in mice. Given the abundance of TF in various cardiac cells (Figure S6), the lower level of TF in the infarcted myocardium may be attributable to decreased TF expression in stressed cardiomyocytes and necrosis of cardiac cells 7. In view of the severe cardiac fibrosis and LV dysfunction following MI, our finding is in line with previous reports that TF expression declines in human failing hearts 7, 8 and TF deficiency causes cardiac fibrosis and LV dysfunction 9. Of note, expression of both TF and its naturally secreted isoform, alternative spliced tissue factor (asTF), which lacks a transmembrane domain due to absence of exon 5, decreases in dilated human hearts 8, and overexpression of asTF in monocytes promotes angiogenesis 35. Whether the structure and stability of asTF produced in TFΔCT mice, in which TF transmembrane domain was removed by deletion of exon 6 10, is not clear. Nonetheless, to our surprise, lack of the TF cytoplasmic domain alleviated post-MI cardiac injury and preserved cardiac function in TFΔCT mice, regardless of gender (Figure S24). These findings suggest that LV dysfunction in TF deficient mice 9 is due to the absence of the TF extracellular domain which contributes to hemostasis rather than the absence of the TF cytoplasmic domain. Deletion of the TF cytoplasmic domain may compensate the TF extracellular domain decline-induced cardiac dysfunction by reducing inflammation and promoting myocardial angiogenesis in MI. Our hypothesis is consistent with the notion that deletion of the TF extracellular domain is lethal while deletion of the TF cytoplasmic domain is viable in mice 10, 11.

The higher survival rate of TFΔCT mice due to less frequent post-MI cardiac rupture indicates that lack of the TF cytoplasmic domain improves cardiac repair, a process crucially involving ECM turnover 27. In response to cardiac injury, MMP2 and MMP9 are activated to disrupt ECM and facilitate infiltration of immune cells to clear necrotic cellular debris 2, 3. However, excess ECM degradation may lead to cardiac rupture 22, 36, 37. Interestingly, lack of the TF cytoplasmic domain did not disturb levels of MMP2 or MMP9 nor the initial clearance of necrotic cardiomyocytes. Instead, it increased TIMP1 expression, and consequently slowed down ECM degradation and inhibited infiltration of phagocytes (neutrophils and macrophages) in infarcted TFΔCT hearts. ECM remodeling is also partly attributable to activation and proliferation of myofibroblasts 38. In this study, we also observed significant proliferation of (myo)fibroblasts following MI, likely due to upregulation of PAR2 39. In addition, we found more proliferative cells and differentiated myofibroblasts as well as higher expression of TGF-β1 in infarcted TFΔCT hearts, indicating a greater reparative response, accompanied by secretion of larger amount of ECM components.

It is of a great importance to note that increased collagen deposition in TFΔCT hearts did not expand beyond the infarct region (Figure 4F). This confinement of collagen deposition ensures a more mature and stable scar whilst not adversely influencing LV remodeling and contractility in the infarcted heart. Furthermore, the resultant larger remaining acellular matrix area in TFΔCT hearts at 7 days post-MI suggests a delay in wound healing. This delay, rather than a complete blockage of cardiac healing as seen in uPA deficient mice 40, appears sufficient to prevent cardiac rupture while not worsen LV remodeling. In fact, post-infarct wound healing in TFΔCT hearts was enhanced at a later stage as evidenced by the better scar formation and smaller scar size at 28 days after MI. Taken together, lack of the TF cytoplasmic domain delays but improves cardiac wound healing following MI with a decrease in cardiac rupture or adverse remodeling.

Deletion of the TF cytoplasmic domain reduces inflammatory response in sepsis-like endotoxemia 13, 14 and liver injury 41. In line with this, our data demonstrate that the TF cytoplasmic domain also mediates inflammatory response to cardiac injury. We used histological staining, instead of FACS analysis, to quantify the spatial and temporal infiltration of various immune cells following MI as the infarcted heart is histologically heterogenous, divided into infarct region, infarct boarder region, and remote region 22, 42. It is worth noting that more neutrophils were observed in the infarct border region of TFΔCT hearts at 7 days post-MI. This observation is unexpected as higher blood counts of neutrophils predicts adverse outcome in MI patients 43. Given the lower levels of neutrophil-secreted pro-inflammatory cytokines, including IL-1β and TNFα, in infarcted TFΔCT hearts, these persistently retained neutrophils might remain inactivated. In fact, deletion of the TF cytoplasmic domain inhibits neutrophil activation and cytokine release in a mouse model of antiphospholipid syndrome 44. Alternatively, those neutrophils may belong to N2-neutrophils (or low-density neutrophils), a subset of neutrophils (up to 18% of total neutrophils infiltrated myocardium at D7 post-MI) contributing to inflammation resolution 45. N2-neutrophils express high levels of anti-inflammatory CD206 and IL-10 and initiate the wound healing in infarcted heart 45, 46. In agreement with our findings, absence of the TF cytoplasmic tail was also reported to attenuate inflammatory response in arthritis 47.

T cells, consistent with previous reports 22, 48, 49, were recruited to the heart following MI and peaked at 3 days in WT mice. However, infiltration of T cells was delayed in infarcted TFΔCT hearts. Considering the better survival and more favorable LV remodeling in TFΔCT mice, one may speculate that those later-appearing T cells might be predominantly Th2 and regulatory T cells, which play protective roles in MI 48-50. The overall myocardial infiltration of monocytes/macrophages following MI was significantly lower in TFΔCT mice. Further analysis demonstrated that more pro-inflammatory M1 macrophages and less reparative M2 macrophages in the infarcted WT hearts, likely because the TF cytoplasmic domain drives macrophage polarization toward an inflammatory M1 phenotype as evidenced in *in vitro* (Figure S15). Given the dynamic polarization of macrophages in the process of post-infarct LV remodeling 51, 52, the change of M1/M2 macrophage proportion in the injured myocardium may contribute to the improved LV remodeling in TFΔCT mice.

Our findings that deletion of the TF cytoplasmic domain suppressed the upregulation of PAR1 and a PAR1 downstream signaling molecule Rac1 in the infarcted myocardium strongly suggest that the TF cytoplasmic domain mediates inflammatory response through PAR1 signaling in the infarcted heart. In fact, the TF cytoplasmic domain induces cytokine production through activation of NF-kB 13, with PAR1 as a key mediator 53. Our hypothesis is supported by the observations that disruption of TF-PAR1 signaling pathway suppresses the inflammatory response in renal injury 53, 54. Moreover, pharmaceutical inhibition of PAR1 55 and PAR1 deficiency 18 ameliorates post-infarct LV remodeling.

In contrast to PAR1, the role of PAR2 in cardiac inflammation is controversial. For instance, cardiomyocyte-specific overexpression of PAR2 was reported to cause cardiac inflammation 56, and PAR2 deficiency attenuated inflammation resulting in smaller infarct size and favorable post-MI LV remodeling 17, 56. Infusion of a PAR2-activating peptide in I/R injury, however, protects cardiac function 19. A recent study by Zhong et al. further confirmed that PAR2 activation protected mouse heart from IR injury via activation of the lipoxygenase pathway and TRPV1 channels 57. More interestingly, Friebel et al. demonstrated that PAR2 deficiency led to increased fibrosis and diastolic dysfunction in heart failure with preserved ejection fraction (HFpEF) 58. Regardless of this controversy, TF was intact in all those prior studies. We now demonstrate that truncating the TF cytoplasmic domain enhances PAR2 expression while attenuating inflammation and post-MI LV remodeling. These findings suggest that, apart from modulating inflammation, PAR2 may benefit the ischemic heart by other mechanisms such as promoting angiogenesis 16 and inhibiting fibrosis in the absence of its negative regulator, the TF cytoplasmic domain.

Cardiac angiogenesis following MI helps local tissue reperfusion (revascularization), leading to better cardiac repair and eventually to more favorable LV remodeling and preservation of cardiac function 4. In this study, regional strain analysis revealed that the overall improvement of cardiac contractile function in TF∆CT hearts was mainly attributed to contractility improvement within the infarct-and-border region rather than the remote myocardium. Accordingly, TF∆CT hearts showed higher vessel densities than WT hearts in the infarct-and-border region but not in the remote region. Therefore, we hypothesize that the favorable cardiac outcomes observed in TF∆CT mice are at least partly attributable to the increased myocardial angiogenesis. In concord, we observed proliferation of endothelial cells in the infarcted myocardium and particularly within the infarct border region, likely due to local proliferation of cardiac resident endothelial cells 59. Importantly, the endothelial proliferation was significantly more pronounced in TF∆CT hearts, explaining the enhanced post-MI myocardial angiogenesis. Furthermore, our data suggest that post-infarct myocardial angiogenesis is mediated by the TF cytoplasmic domain via a PAR2- but not PAR1-dependent pathways. Similar to ocular angiogenesis in diabetes 16, 60, post-infarct myocardial angiogenesis is regulated by the TF cytoplasmic domain-PAR2 angiogenic axis likely via PDGF-B- rather than VEGF-related signaling pathways. Furthermore, we also demonstrated that other key angiogenic pathways including DLL4-NOTCH and SDF-1α-CXCR4 were involved in post-infarct angiogenesis and modulated by the TF cytoplasmic domain. Given that all the five pro-angiogenic factors influenced by the TF cytoplasmic domain were mainly produced from cardiac endothelial cells (Figure S23), the higher levels of those proangiogenic factors likely resulted from the enhanced proliferation of endothelial cells in TF∆CT hearts (Figure 3).

Bone marrow transplantation allows to delineate function of the cells of hematopoietic origin and estimate the relative contribution of hematopoietic stem cells and resident cells in animal models 22, 24, 61. Since TF is highly expressed in both circulating monocytes/macrophages (of hematopoietic origin), cardiomyocytes and fibroblasts (cardiac resident cells), we tried to identify the cellular source of TF contributing to post-infarct LV remodeling by cross-over bone marrow transplantation. Although transplantation of TF∆CT bone marrow cells into WT mice resulted in better heart function compared to TF∆CT mice receiving WT bone marrow (LVEF: 30.7 ± 1.7 vs. 26.1 ± 1.8), the difference did not reach statistical significance. These results indicate that both cardiac resident cells and infiltrating cells of hematopoietic origin contribute to post-infarct LV remodeling even though cells from bone marrow appears to have a dominant role. In fact, hematopoietic stem cells do not only give rise to myeloid cells but also to fibroblasts (25%) and myofibroblasts (57%) in the infarcted myocardium 62. In the infarcted heart up to 17% of collagen is produced from fibroblasts of hematopoietic origin 63. Therefore, the partial replacement of TF expressing cells in bone marrow chimeric mice makes it difficult to delineate specific cell type(s) that contribute to the TF cytoplasmic domain-mediated signaling in the infarcted heart.

Our findings also raise several open questions. For instance, the ligands that activate TF intracellular signaling pathways in MI remain elusive. Given that absence of the TF cytoplasmic domain in either cardiac resident cells or bone marrow cells alleviated post-MI cardiac function and remodeling, how the TF cytoplasmic domain-PAR1-Rac1 axis and -PAR2-angionenic pathways are coordinated among specific cell types to yield the favorable outcome in TF∆CT mice needs further study. Nonetheless, the recapitulation of WT mouse phenotype by activating PAR1 while inhibiting PAR2 activity in TF∆CT confirms that the TF cytoplasmic domain exacerbates post-infarct LV remodeling via differential regulation of PAR1 and PAR2. Yet, whether TF directly interacts with the two PARs or via certain adaptor proteins such as Rac1 in the context of intracellular signaling remains unclear.

In conclusion, the TF cytoplasmic domain orchestrates pathological inflammation and angiogenesis via synergistic regulation of PAR1 and PAR2 activities in the infarcted heart thereby contributing to adverse post-infarct LV remodeling. Earlier studies have shown that functional blockage of TF by disrupting the interaction between the TF extracellular domain and coagulation factor VII or thrombin is cardioprotective in MI 64-66, however, the strategy potentially leads to cardiac hemorrhage, thus impeding the clinical translation. Our findings suggest that targeting the cytoplasmic domain of TF may be an attractive therapeutic strategy for post-infarct cardiac repair and LV remodeling without perturbing TF coagulation function.

## Supplementary Material

Supplementary materials and methods, figures, and tables.

Supplementary video 1: Baseline_WT.

Supplementary video 2: Baseline_TFdeltCT.

Supplementary video 3: MI_WT.

Supplementary video 4: MI_TFdeltCT.

## Figures and Tables

**Table 1:** Echocardiography characterization

	WT			TF∆CT		
	Baseline	7 days MI	28 days MI	Baseline	7 days MI	28 days MI
HR (bpm)	397.9 ± 7.0	444.7 ± 12.2‡	428.2 ± 14.9	391.5 ± 9	444.5 ± 21.2	385.6 ± 8.6§
ESV (μl)	31.92 ± 1.7	95.10 ± 11.5‡	114.5 ± 13.1‡	28.32 ± 1.5	66.91 ± 13.97*	67.06 ± 10.8†#
EDV (μl)	65.51 ± 2.6	124.7 ± 10.9‡	146.6 ± 12.4‡	61.01 ± 2.1	94.35 ± 13.4*§	99.97 ± 11.71†#
EF (%)	51.25 ± 1.5	27.4 ± 2.9‡	24.69 ± 2.5‡	53.88 ± 1.2	33.00 ± 3.413‡	35.91 ± 1.93‡#
FS (%)	26.11 ± 1.0	11.37 ± 1.3‡	10.44 ± 1.2‡	28.69 ± 1.5	12.63 ± 1.7‡	13.03 ± 1.3‡
LVIDs (mm)	3.086 ± 0.08	4.609 ± 0.24‡	5.072 ± 0.24‡	2.875 ± 0.1	4.265 ± 0.31‡	4.326 ± 0.25‡§
LVIDd (mm)	4.158 ± 0.06	5.158 ± 0.19‡	5.622 ± 0.2‡	4.011 ± 0.07	4.846 ± 0.26‡	4.938 ± 0.21‡#

Echocardiography was performed on the mice at baseline (before MI) and at the indicated time after MI. Paired t-test compared to respective baseline: *p < 0.05, †*p* < 0.01, ‡*p* < 0.001; Mann-Whitney U test compared to WT: §*p* < 0.05, #*p* < 0.001. N = 19 for WT mice and n = 19 for TF∆CT mice. HR, heart rate (beat per minute); ESV, end systolic volume; EDV, end diastolic volume; EF, ejection fraction; FS, fractional shortening; LVIDs, left ventricular internal diameter end systole; LVIDd, left ventricular internal diameter end diastole.

## Abbreviations

BM: bone marrow
ECM: extracellular matrix
IL: interleukin
LV: left ventricle
MI: myocardial infarction
MMP: matrix metalloproteinase
PAR: protease-activated receptor
PDGF: platelet derived growth factor
TF: tissue factor
TIMP-1: tissue inhibitor of metalloproteinase 1
VEGF: vascular endothelial growth factor
WT: wild type
