# Supplementary material for: Tissue factor cytoplasmic domain exacerbates post-infarct left ventricular remodeling via orchestrating cardiac inflammation and angiogenesis
Source: Theranostics. 2021 Sep 3;11(19):9243–61. doi: 10.7150/thno.63354 (PMC8490508; doi:10.7150/thno.63354)
Supplement: Supplementary file 1 — Supplementary materials and methods, figures, and tables. [file thnov11p9243s1.pdf]

SUPPLEMENTAL MATERIAL

Tissue factor cytoplasmic domain exacerbates post-infarct left ventricular remodeling via orchestrating cardiac inflammation and angiogenesis

Suet Yen Chong[1,2*], Olga Zharkova[1,2*], Siti Maryam J.M. Yatim[1,2], Xiaoyuan Wang[1,2], Xiong Chang Lim[2], Chenyuan Huang[1,2], Chia Yee Tan[2,3], Jianming Jiang[2,3], Lei Ye[4], Michelle Siying Tan[1], Veronique Angeli[5,6], Henri H. Versteeg[7], Mieke Dewerchin[8,9], Peter Carmeliet[8,9], Carolyn S.P. Lam[10,11], Mark Y. Chan[2,12], Dominique P.V. de Kleijn[13,14], Jiong-Wei Wang[1,2,15,16]

[1]Department of Surgery, Yong Loo Lin School of Medicine, National University of Singapore, Singapore, Singapore
[2]Cardiovascular Research Institute (CVRI), Cardiovascular Disease TRP, National University Heart Centre Singapore (NUHCS), Singapore, Singapore
[3]Department of Biochemistry, Yong Loo Lin School of Medicine, National University of Singapore, Singapore, Singapore
[4]National Heart Research Institute Singapore, National Heart Centre Singapore, Singapore.
[5]Immunology translational research program, Department of Microbiology and Immunology, Yong Loo Lin School of Medicine, National University of Singapore, Singapore,
[6]Immunology Program, Life Sciences Institute, National University of Singapore, Singapore
[7]Einthoven Laboratory for Experimental Vascular Medicine, Leiden University Medical Centre, The Netherlands
[8]Laboratory of Angiogenesis and Vascular Metabolism, Center for Cancer Biology (CCB), VIB, Leuven, Belgium
[9]Laboratory of Angiogenesis and Vascular Metabolism, Department of Oncology and Leuven Cancer Institute (LKI), KU Leuven, Leuven, Belgium
[10]National Heart Centre Singapore (NHCS), Duke-NUS Graduate Medical School, Singapore, Singapore
[11]Department of Cardiology, University Medical Center, Groningen, The Netherlands
[12]Department of Medicine, Yong Loo Lin School of Medicine, Singapore, Singapore
[13]Netherlands Heart Institute, Utrecht, the Netherlands
[14]Department of Vascular Surgery, University Medical Center Utrecht, Utrecht, The Netherlands
[15]Department of Physiology, Yong Loo Lin School of Medicine, National University of Singapore, Singapore, Singapore
[16]Nanomedicine translational research programme, Centre for NanoMedicine, Yong Loo Lin School of Medicine, National University of Singapore, Singapore, Singapore
* These authors contributed equally

Address correspondence to:

Jiong-Wei Wang, PhD

Department of Surgery, Cardiovascular Research Institute

National University of Singapore

14 Medical Drive, MD6, #08-01, 117599, Singapore

Tel: +65 66011387

Email: surwang@nus.edu.sg

SUPPLEMENTAL MATERIALS AND METHODS:

Animals: All mice used for this study were of C57/BL6Jax background. TFΔCT mice, which lack the 18 carboxy-terminal intracellular amino acids of TF were obtained from University of Leuven [1]. The genotype of TFΔCT mice was confirmed by PCR (*Figure S1*). Wild-type (WT) C57/BL6 mice from Jackson laboratories were purchased from InVivos (Singapore). Male mice, unless stated otherwise, at 8 - 12 weeks old and 20 - 25 g were used for all experiments. Animals were given irradiated standard laboratory diet 2918 (ENVIGO) and water *ad libitum*, and housed under specific pathogen-free (SPF) conditions with a 12/12-hour light-dark cycle (lights on at 7 AM, lights off at 7 PM) at the Comparative Medicine Animal Vivarium in the National University of Singapore. All studies were approved by the National University Singapore Institutional Animal Care and Use Committee (IACUC) and conformed to the guidelines on the care and use of animals for scientific purposes (NACLAR, Singapore, 2004) and the Guide for the Care and Use of Laboratory Animals published by the US National Institutes of Health (NIIH Publication, 8th Edition, 2011).

Myocardial Infarction Animal Model: MI was induced in WT or TFΔCT mice (10-12 weeks old, 20-25 g) as previously described [2, 3]. In brief, mice were anesthetized by one i.p. injection of a mixture of 0.5 mg/kg medetomidine, 5.0 mg/kg dormicum and 0.05 mg/kg fentanyl. MI was induced by permanent occlusion of the left anterior descending coronary artery (LAD). Mouse body temperature was monitored with a rectal probe and maintained at 37°C during surgery. After surgery, mice were recovered by a subcutaneous injection of 0.5 mg/kg atipamezole and 5 mg/kg flumazenil, and received 0.1 mg/kg buprenorphine subcutaneously twice daily for 3 days. Mouse heart tissue and blood were harvested at predetermined endpoints after euthanasia with an overdose of ketamine (225 mg/kg) and medetomidine (3 mg/kg) intraperitoneally.

Infarct size was determined 24 hours after MI using Evans Blue and triphenyltetrazolium chloride (TTC) staining as previously described [4]. Plasma levels of Troponin was determined by ELISA (LifeDiagnostics, PA, USA) according to the manufacturer's instruction.

The surgeon was blinded to mouse genotypes during the procedures. Mice that died prior to completion of the study protocol were used to determine mortality rates but excluded from other analyses.

Bone Marrow Transplantation: Chimeric mice were generated as previously described [5]. In brief, bone marrow (BM) cells were collected from WT (CD45.1) and TFΔCT (CD45.2) mice with RPMI-1640 medium (ThermoFisher, MA, USA). Recipient mice received 5x10^6 BM cells after receiving a single dose of 7Gy radiation from a Biobeam 8000 (137Cs source) irradiator (Gamma-Service Medical GmbH, Leipzig, Germany). After 6 weeks, chimerization was confirmed by flow cytometry analysis of peripheral blood stained with the CD45.1 conjugated to PerCP-Cy5.5 (Clone A20) and CD45.2-FITC (Clone 104) (eBioscience) using BD LSR Fortessa (BD, NJ, USA). The Isotype controls and Fluorescence Minus One Controls (FMO) were used to define gates and interpret flow cytometry data. Data were analyzed using FlowJo (BD) software (v10.1). Transplantation was considered successful with more than 95% leukocytes derived from donor BM (*Figure S2*). Recipient WT mice receiving TFΔCT BM were referred as WT/TFΔCT BM mice and recipient TFΔCT mice receiving WT BM as TFΔCT/WT BM mice.

Echocardiography: Cardiac function was assessed with a high frequency ultrasound system Vevo® 2100 (FUJIFILM VisualSonics, The Netherlands) as previously described [3]. Briefly, echocardiography was performed on mice under general anesthesia (inhalation of 1-1.5% isoflurane, Baxter, Singapore). Body temperature was monitored with a rectal probe and maintained at 37°C during imaging. Cardiac volumes and functional parameters were derived from parasternal long-axis B-mode images (LV trace mode). Speckle-tracking strain analysis (radial strain) was performed on long-axis B-mode images over three consecutive cardiac cycles with the VevoStrain software (Visualsonics) as previously described [4]. Radial strain and strain rate were analyzed based on the semiautomated endocardial and epicardial border detection in a parasternal long-axis view (*Figure S6A*). According to the LV anatomy and the location of infarct, we assigned corresponding segments to infarct-and-border and remote regions. All the data were analyzed with Vevo® 2100 software (version 1.7.0) by an experienced researcher who was blinded to the mouse genotypes and experimental procedures.

Minipump delivery system: Osmotic minipump (Alzet 2004 - Rate: 0.25 µl/hr; Durect, Cupertino, CA) implantation was performed subcutaneously through skin incision before MI surgery as we previously described [6]. Prior to implantation osmotic pumps were filled with 200 ul cocktail of PAR1 agonist peptide (TFLLR-NH2) and PAR2 antagonist peptide (FSLLRY-NH2), or a control peptide (RLLFT-NH2; all peptides TOCRIS), at concentration of 1 mg/ml and

calibrated 40 h at 37 ºC in PBS. Minipumps were charged to deliver peptides continuously at 3 mg·kg^{-1}·day^{-1} for 28 days.

Cell Isolation, Culture and Sorting: Isolation of cardiac resident cells including cardiomyocytes, fibroblasts, endothelial cells and resident immune cells, and culture of BM-derived macrophages were described previously [3]. In brief, 8-12 weeks old WT mice were anesthetized with isoflurane followed by cut of the inferior vena cava. After intensive perfusion with EDTA buffer (5mM) and collagenase buffer (collagenases II and IV, and protease XIV), the left ventricle was dissociated into small pieces and forced to pass through a cell strainer (100µm pore-size). After 30 min of gravitational sedimentation, the supernatant containing the non-cardiomyocytes population were transferred to a new falcon tube while the remaining cell pellets were collected and re-suspended in calcium-reintroduction buffer prior to plating into a 6-well plate pre-coated with murine laminin (ThermoFisher). The non-cardiomyocytes and dead cells were washed and removed after one hour and the attached cardiomyocytes were incubated in the M199 culture medium (MilliporeSigma, Missouri, USA) for subsequent experiments.

The supernatant collected earlier containing cardiac fibroblasts, endothelial cells and resident immune cells were centrifuged at 300g for 5 min, and the cell pellets were re-suspended in pre-equilibrated DMEM/F12 medium (ThermoFisher). To culture fibroblasts, half of the resuspended cells were plated on petri dishes, culture medium was refreshed after 24 hours and subsequently every two days. After one week of culture, cells were stained and sorted with MoFlo XDO cell sorter (Beckman Coulter). Podoplanin^{+}/CD45^{-}/DAPI^{-} cell population was collected and plated for immunofluorescence staining. To isolate cardiac resident immune cells the other half of the re-suspended cells collected after cardiomyocytes isolation were stained with antibodies and sorted with SY3200 sorter (SONY). Cardiac resident immune cells (majority are macrophages) defined as CD45^{+}/CD31^{-}/DAPI^{-}. Apart from the isotype control antibodies, the Fluorescence Minus One Controls (FMO) were used to define gates and interpret flow cytometry data. Data were analyzed using FlowJo (BD) software (v10.1).

To isolate cardiac endothelial cells, murine hearts were perfused, minced and digested for 30 min at 37 ºC, with a cocktail of collagenase 2 (Worthington) and DNAse I (Roche), filtered through 70 µm filters and spun down at 450 g, 5 min, 4 ºC. Cardiomyocytes were removed by gravity and the rest of the cells were plated on collagen, for endothelia cells to attach. After

incubation for 3 hours at 37 ºC, plates were washed and fixed. Attached endothelial cells were stained with IB4, TF and DAPI.

Bone marrow cells were cultured in Dulbecco's Modified Eagle Medium (DMEM) supplemented with 20% fetal bovine serum (FBS) (ThermoFisher) and 30 ng/ml M-CSF (ThermoFisher) for 6 days to obtain bone marrow derived macrophages (BMDM). To polarize macrophages towards pro-inflammatory M1 phenotype, BMDM were incubated 24 h with 20 ng/ml LPS and 10 ng/ml IFN-ɣ.

Cell Staining and Imaging: Cardiomyocytes and sorted fibroblasts were fixed and stained after 24h of culture. BM-derived macrophages were stained after one week. Endothelial cells and cardiac resident immune cells were stained directly after sorting. Briefly, the cells were washed twice with ice-cold phosphate buffered saline (PBS) prior to fixation with 10% ice-cold formalin (MilliporeSigma). Cells were incubated with blocking solution containing 0.1% saponin (MilliporeSigma), 5% FBS and 5% bovine serum albumin (BSA) in PBS for one hour at room temperature followed by subsequent incubation with primary antibodies (or respective isotype controls) at 4°C overnight, fluorescent-conjugated secondary antibodies at room temperature for 1 hour and a further 10 min with 250 ng/ml 4',6-diamidino-5-phenylindole (DAPI; ThermoFisher). Stained endothelial cells and cardiac resident immune cells were plated on pre-coated glass slides using Cytofuge 2 cytocentrifuge (Beckman Coulter) for 10 min at 850 rpm, air dried for 3 min and mounted with Vectashield Antifade Mounting Medium with DAPI (VectorLab). Images were taken by a fluorescence microscope (Nikon Eclipse Ti-E inverted microscope, Nikon Instrument Inc., Tokyo, Japan). All the antibodies were listed in *Table S1*.

RNA Isolation and qRT-PCR: Total RNA was extracted from infarct and remote myocardium using RNeasy Mini Kit (QIAGEN, Venlo, The Netherlands) following manufacturer's protocol. Total RNA concentration was determined using NanoDrop 2000 spectrophotometer (ThermoFisher) and 125 ng was used to convert to cDNA using QuantiTect-Reverse-Transcription Kit (QIAGEN). qPCR was then performed in triplicate with iTaq Universal SYBR Green Supermix (Bio-Rad, Hercules, CA, USA) in real-time thermal cycler, QuantStudio 7 (ThermoFisher). GADPH was used as the housekeeping gene and all mRNA expression levels were normalized to sham. The primers used for qRT-PCR were listed in *Table S2*.

Quantification of Inflammatory Cytokines and Chemokines: Multiplex immunoassay (Bio-Plex Pro Mouse Cyokine 23-Plex, Bio-Rad) was used to determine the concentrations of

inflammatory cytokines and chemokines in the infarct and remote myocardium on a Bio-Plex 200 multiplex suspension array system (Bio-Rad). Protein lysates were diluted fifteen times prior to measurement. Concentrations of analytes were normalized to total protein concentration and were expressed as pg/mg protein.

Matrix Metalloproteinase Zymography: Total proteins were extracted as described previously. Tissue lysate containing 5 µg of proteins was mixed with 1X Novex Tris-Glycine SDS Sample Buffer and loaded in Novex Zymogram Gels. Gels were run in a Mini-Cell tank with 1X Tris-Glycine SDS Running buffer for 90 minutes at 125V. 10µL of Spectra Multicolor Broad Range Protein Ladder and recombinant MMP2 and MMP9 were used as the references. Gels were then washed with 1X Novex Zymogram Renaturing Buffer and incubated in 1X Novex Zymogram Developing Buffer overnight prior to staining using SimplyBlue SafeStain (all from ThermoFisher). The gel was visualized with ChemiDoc Touch Gel Imaging System (Bio-Rad) and quantified based on intensity using ImageLab Software (Bio-Rad).

Picrosirius Red Staining: To quantify the density and types of collagen in the infarct myocardium, heart tissue embedded in paraffin was sectioned (5 µM thick) and stained with Picrosirius Red [5]. Images were taken in bright field and circularly polarized filter on a Nikon Eclipse Ti-E inverted microscope (Nikon Instrument Inc.). The types of collagen, which appeared different in color under circularly polarized filter, were quantified using color binary threshold [7] in Nikon AR element analysis software version 4.5.0 (Nikon Instrument Inc.,). The optical properties of collagen fibers change with maturity: the immature and fibrillar fibers appear green in color, whereas mature and thick fibers appear yellow-orange and the most mature ones appear red in color [7, 8]. Collagen content was presented as a percentage of the area of infarct tissue.

Immunohistostaining: Snap-frozen or paraffin-embedded left ventricles were sectioned into 5 µm thick. The list of antibodies used for staining can be found in *Table S1*. Antigens were retrieved for paraffin-embedded tissue with heat-mediated method in Tris-EDTA buffer (pH 9.0). Endogenous peroxidase was blocked with Bloxall solution (Vector Laboratories, Burlingame, Ca, USA). Heart tissue was then incubated with primary antibodies or isotype control antibodies: CD142 (ThermoFisher) for total tissue factor, Ly6G (BD Pharmingen, Heidelberg, Germany) for neutrophils, MAC3 (BD Pharmingen, Heidelberg, Germany) for macrophages, CD3 (Dako, Glostrup, Denmark) for T cells, Ki67 (ThermoFisher) for proliferative cells, aSMA (Abcam) for activated myofibroblasts, PAR2 (ThermoFisher) for PAR2 expressing cells, and biotinylated

Griffonia Simplicifolia Lectin I (GSL I) isolectin B4 (IB4) for vascular endothelial cells, iNOS (Abcam) for inducible nitric oxide synthase enzyme, CD206 (Bio-Rad, Hercules, California, US) for mannose receptor, CD68 (Bio-Rad, Hercules, California, US) for macrophages, or isotype control IgG (Abcam), followed by appropriate secondary antibodies conjugated with HRP or fluorescent dye. NovaRED Peroxidase (HRP) Substrate kit was used to visualize the staining according to manufacturer's instruction (Vector Laboratories). All sections were counterstained with hematoxylin or DAPI to visualize the cell nucleus. Some sections were co-stained with wheat germ agglutinin conjugated with FITC (WGA; Vector Laboratories) to indicate the tissue morphology. Images were taken in bright field or fluorescent channel and were analyzed using NIS-Element AR Analysis software version 4.5 (Nikon Instruments Inc.). Inflammatory cells and Ki67 positive cells were quantitated in the whole ventricle area by semi-automated binary threshold detection method and the results were presented as number of cells per mm^2 of tissue and percentage of positive cells of total tissue area, respectively. aSMA, PAR2 and angiogenesis were quantitated using the same method but presented as a percentage of positive staining of total tissue area. Co-staining of CD68 with either iNOS or CD206 represented M1 and M2 subtype macrophages and were expressed as percentage over total $CD68^+$ cells.

Western blot: Total proteins were extracted from heart tissue with RIPA buffer supplemented with protease and phosphatase inhibitor cocktail (ThermoFisher). Twenty ug protein per sample was fractionated by SDS-PAGE and transferred to nitrocellulose membrane using iBlot 2 Dry Blotting System (ThermoFisher). Following blocking with 5% non-fat milk in TBST buffer, the membranes were probed with primary antibodies (*Table S1*) at 4°C for 16h. Then, the membranes were washed 3 times (5min/time) followed by incubation with horseradish peroxidase-conjugated secondary antibodies for 1h at room temperature. Blots were washed 4 times (5min/time) and developed with ECL substrates (MerckMillipore) according to manufacturer instructions. Blots were visualized with ChemiDoc Imager and quantified with ImageJ software.

Statistical Analysis: Statistical analysis was performed using SPSS software (IBM® SPSS® Statistics version 22.0). Gaussian distribution was assessed, and the appropriate Student's t-test or Mann-Whitney U test was used to compare two groups, with one-way ANOVA or two-way repeated measures ANOVA followed by Bonferroni *post hoc* testing of multi-group comparisons. Kaplan-Meier analysis with log rank testing was used for inter-group comparisons

of mortality. Sample sizes are indicated in the figure legends. Heatmaps for cytokines and chemokines were generated by Prism 8.0 software (GraphPad). Data are presented as mean ± SEM. $P < 0.05$ is considered statistically significant.

SUPPLEMENTAL FIGURES

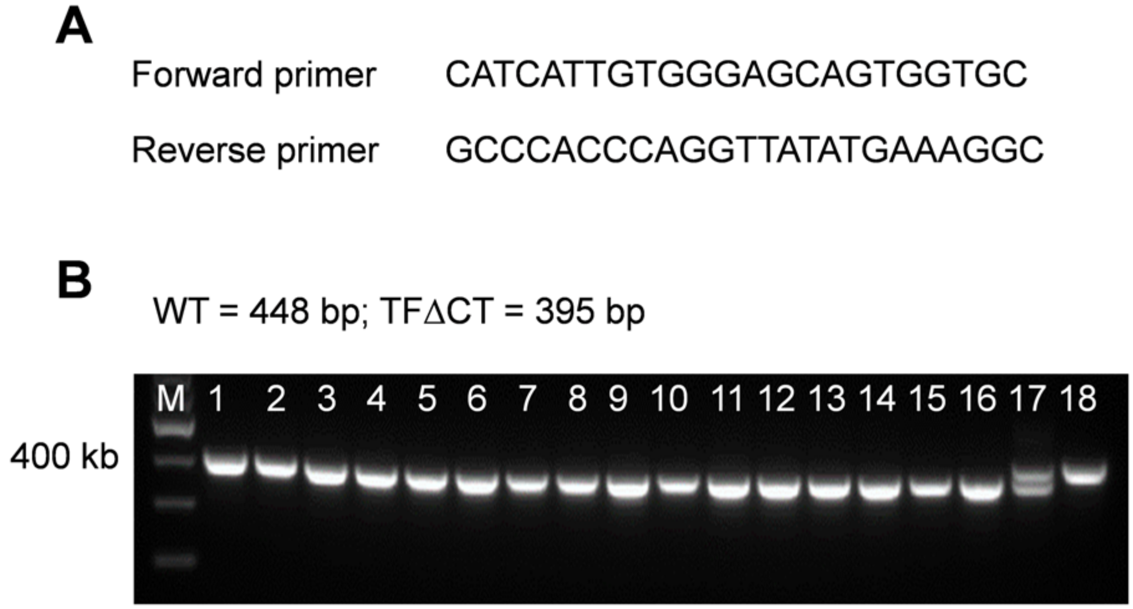

Figure S1. TFΔCT mice genotyping. A, Primers for PCR. **B**, Gel electrophoresis of PCR product: lane 1-16, TFΔCT mice; lane 17, heterozygous mouse; and lane 18, WT mouse**.**

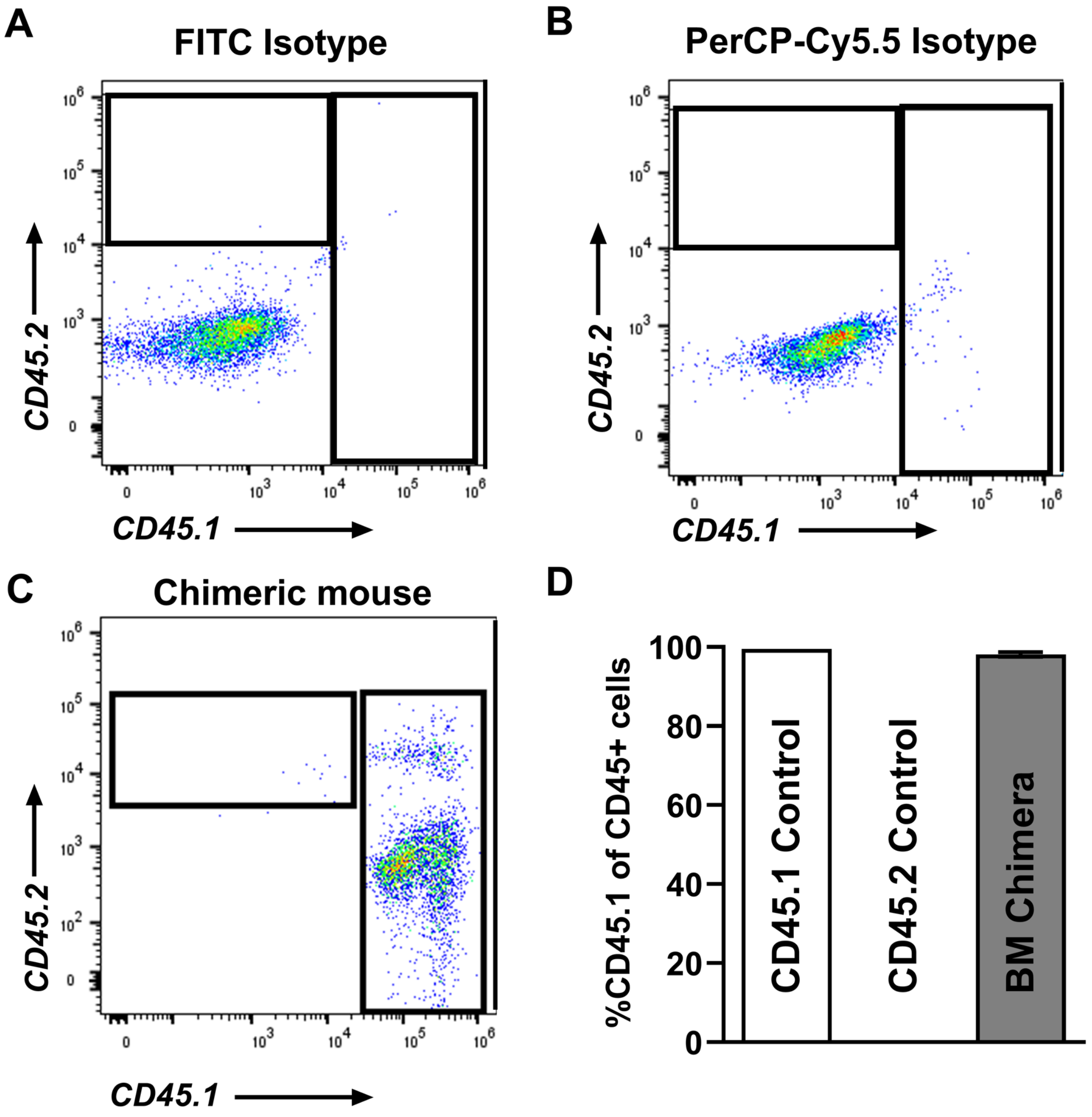

Figure S2. Confirmation of chimerism in mice following bone marrow transplantation. Representative FACS plots for mouse whole blood stained with (**A**) FITC isotype control and (**B**) PerCP-Cy5.5 isotype control. **C**, Representative FACS plots from chimeric mouse whole blood stained with CD45.1 and CD45.2 antibodies. **D**, Percentage of CD45.1 leukocytes out of total $CD45^+$ cells in blood. In the chimeric mice (CD45.2 mice receiving CD45.1 bone marrow) more than 97% of $CD45^+$ cells were of CD45.1 origin. N = 5 for BM chimeras and n = 1 per control. Data were analyzed using FlowJo (BD) software (v10.1).

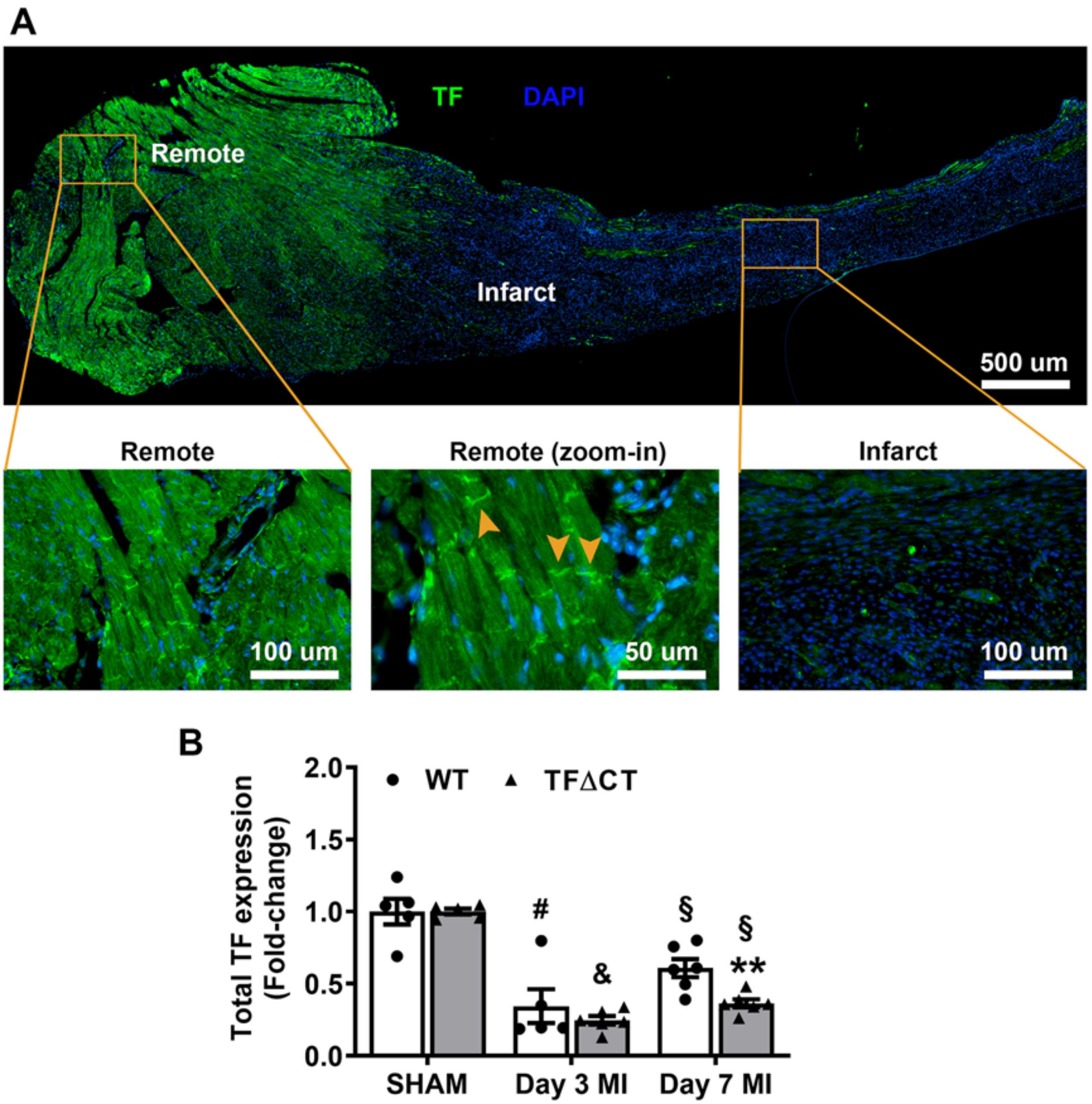

Figure S3. TF expression decreases in the infarcted myocardium. A, A representative heart section showing TF spatial distribution following MI. Arrowheads indicate TF at the intercalated discs in the remote myocardium. TF was stained and visualized by Alexa Fluor 594 (converted to pseudo green color). **B,** mRNA levels of total TF were determined in infarcted heart tissue at 3 and 7 days post-MI. The mRNA level was normalized to GAPDH and presented relative to a genotype match sham set as 1 (n = 5 - 6 per genotype per time-point). Mann–Whitney U test, $^{**}p < 0.01$ compared with WT mice; $\#p < 0.01$, $\&p < 0.001$ compared with sham; $\S p < 0.01$ compared day 3 post-MI.

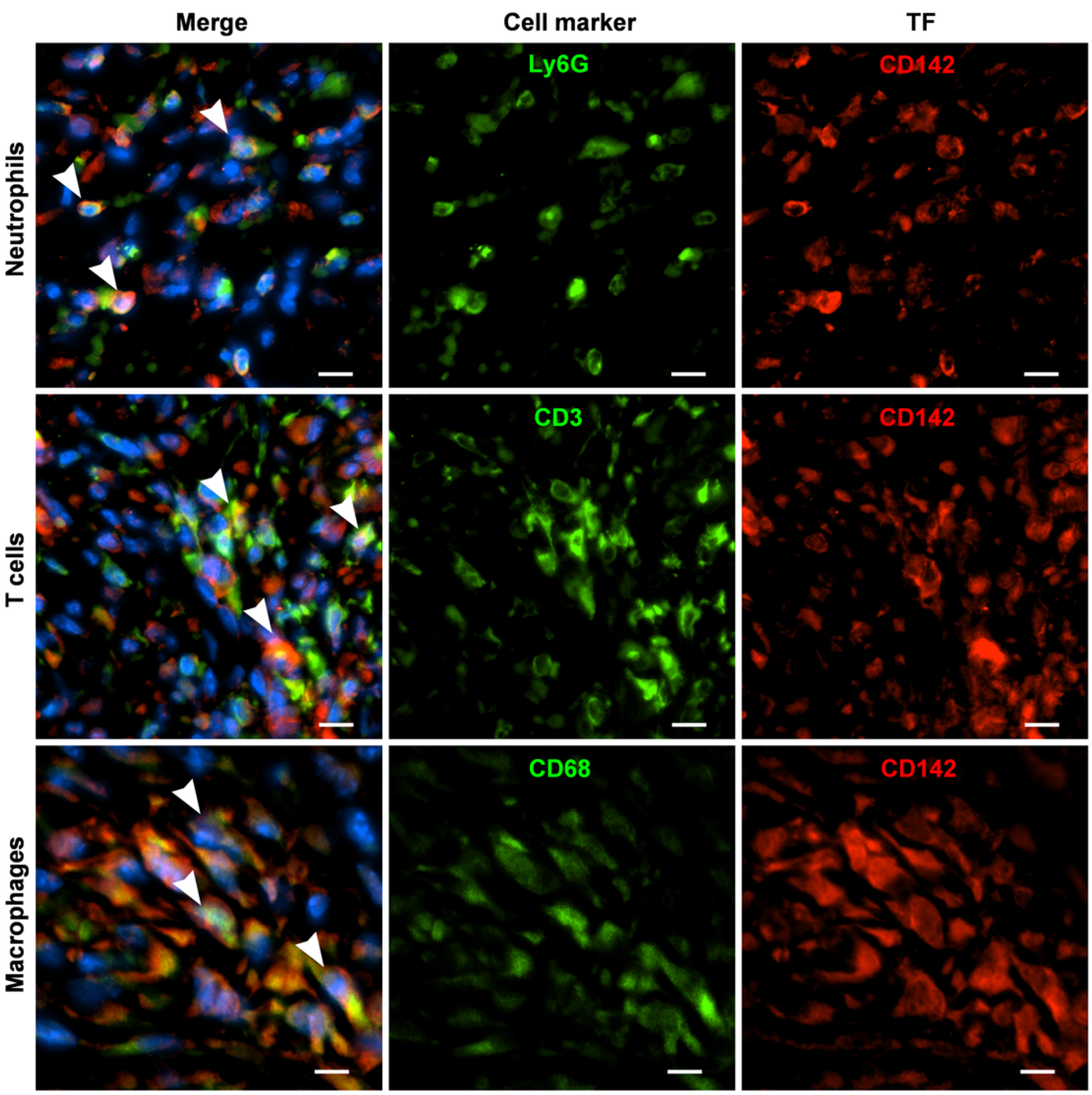

Figure S4. TF expression in different immune cells in the infarcted myocardium. Representative immunofluorescence staining of neutrophils in WT hearts at day 3 post-MI while T-cells and macrophages were stained in WT hearts at day 7 post-MI. Cell specific markers: Ly6G for neutrophils, CD3 for T-cells and CD68 for macrophages. All tissues were stained together with TF antibody CD142. Alexa Fluor-488 and Alexa Fluor-594 were used as secondary antibodies. DAPI was used to visualize the cell nuclei. White arrowheads indicate co-localization of immune cell markers with TF. Scale bars = 10 µm.

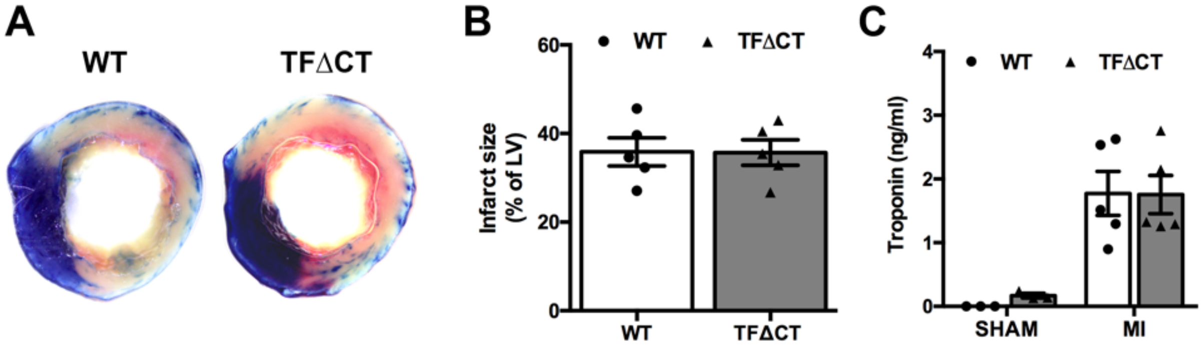

Figure S5. Initial infarct size in MI induced by permanent occlusion of LAD. A, Representative heart slices illustrating the infarct area in pale and non-infarct area in blue (examined by Evan's blue-TTC staining 24 hours post-MI). **B,** Infarct size (same as area at risk in this MI model) was quantified and presented as % of LV. **C,** Cardiac muscle damage induced by MI was also determined with troponin I test in mouse plasma. Each bar represents mean ± SEM, n = 5 per group, Mann–Whitney U test.

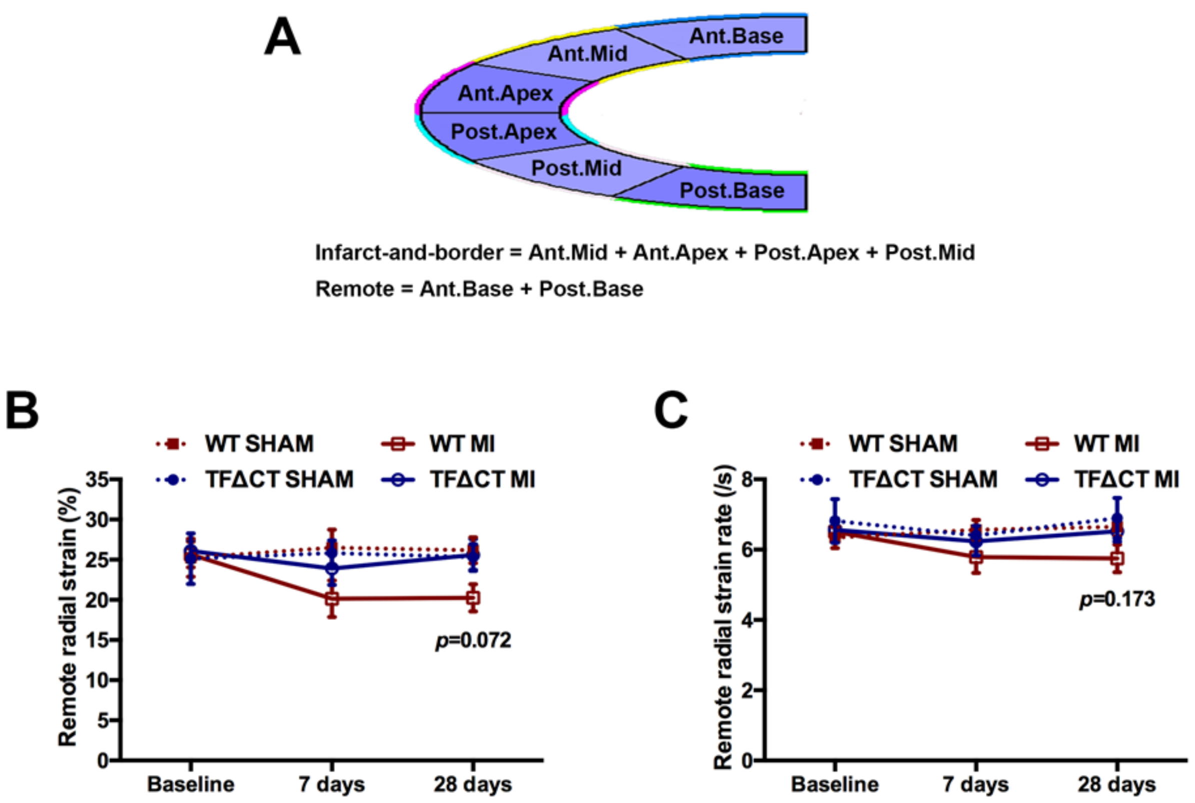

Figure S6. **Cardiac contractility and myocardium deformation in the remote region. A**, A schematic diagram illustrating global and regional strain analysis. Radial strain (**B**) and radial strain rate (**C**) of the infarct remote regions in MI hearts at baseline, 7 and 28 days after MI. *P* values indicate difference between TFΔCT and WT MI hearts; n = 19 for WT mice and n = 17 for TFΔCT mice subjected to MI, n = 10 for WT and n = 9 for TFΔCT sham groups; two-way repeated measures ANOVA followed by Bonferroni *post hoc* testing. Ant.Base, anterior base; Post.Base, posterior base, Ant.Mid, anterior middle; Post.Mid, posterior middle; Ant.Apex, anterior apex; Post.Apex, posterior apex.

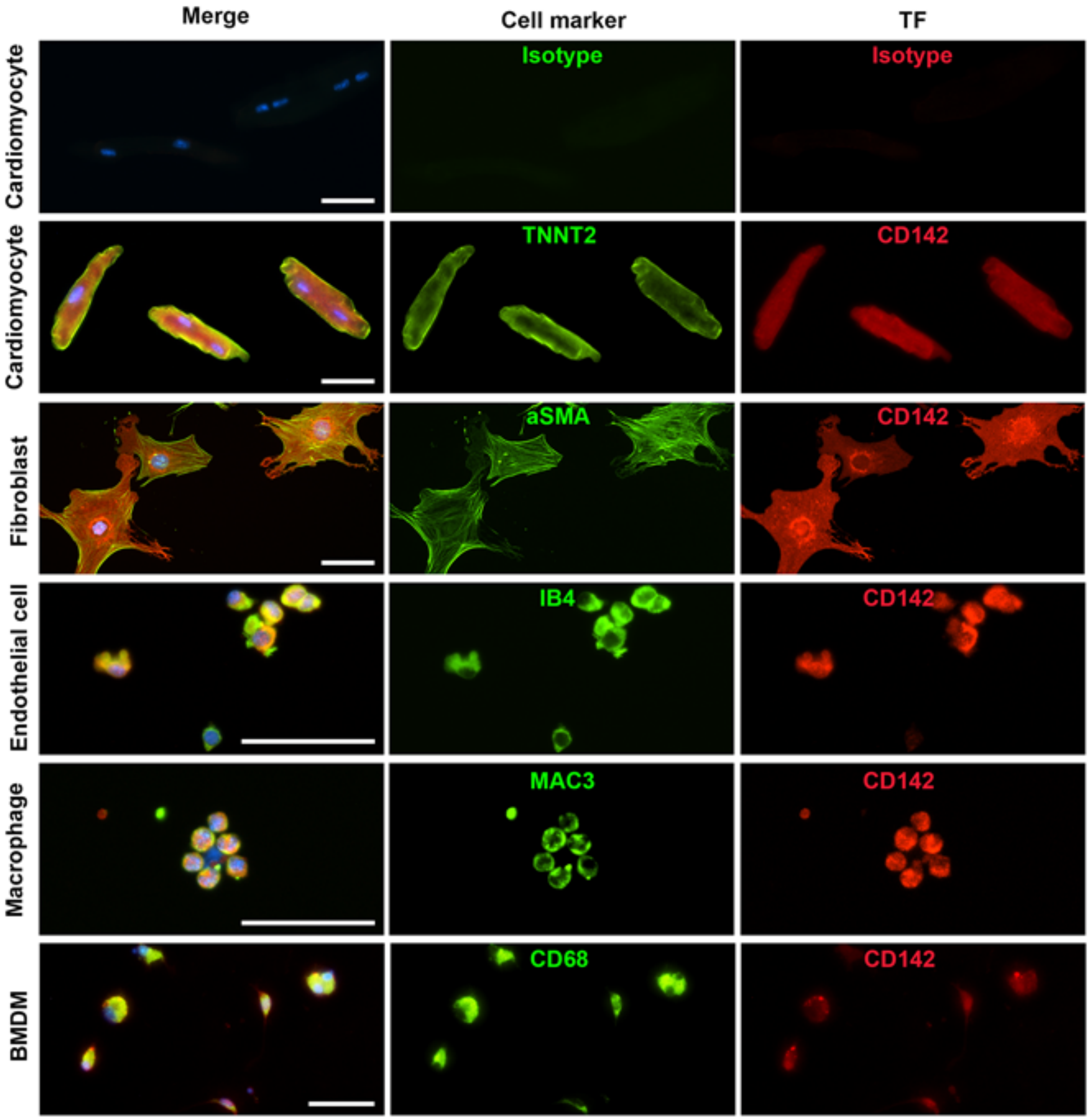

Figure S7. **Expression of TF in different cell types.** Cardiomyocytes, fibroblasts, endothelial cells and resident macrophages were isolated from adult mouse hearts, and bone marrow-derived macrophages (BMDM) were derived from bone marrow. Cardiac endothelial cells and resident macrophages were sorted after isolation and plated on glass slides using a cytospin. Cell specific markers: cardiac troponin-T, isoform Ab-1 (TNNT2) for cardiomyocytes; aSMA for fibroblasts; griffonia simplicifolia lectin I (GSL I) isolectin B4 (IB4) for endothelial cells and CD68 for BMDM. All cells were sequentially stained with tissue factor antibody CD142. Isotype controls were used to verify antibody staining in cardiomyocytes (top row) and in all the other four types of cells (not shown). Alexa Fluor-488 and Alexa Fluor-594 were used as secondary antibody. DAPI was used to visualize the cell nuclei. Scale bars = 50 μM.

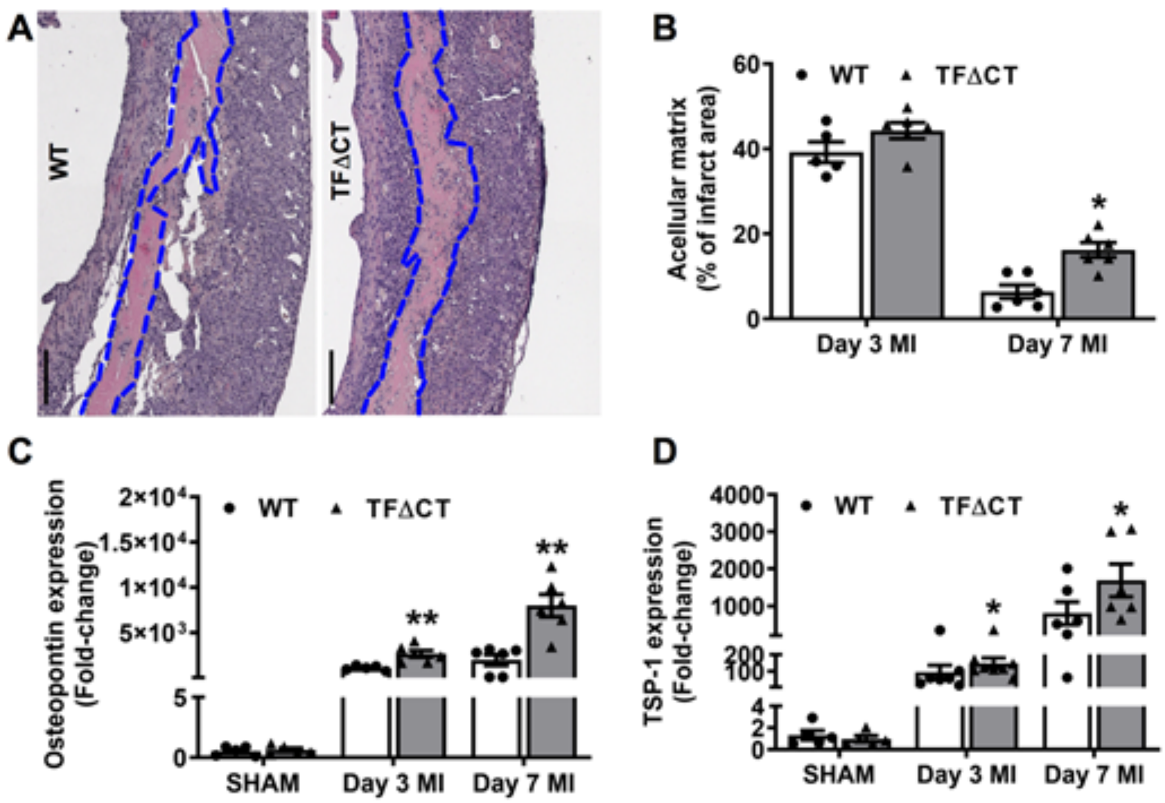

Figure S8. **Acellular matrix areas and expression of extracellular matrix proteins. A,** Representative images of acellular matrix area in the infarct regions in WT and TFΔCT mice at 7 days post-MI. **B,** Quantitation of acellular matrix areas. N = 5 - 6 per genotype per time-point. **C** and **D,** mRNA expression of osteopontin and thrombospondin 1 (TSP-1) in the heart tissue of WT and TFΔCT mice (n = 5 - 6 per genotype per time-point). Data represent mean ± SEM, Mann–Whitney U test, *$p < 0.05$, **$p < 0.01$.

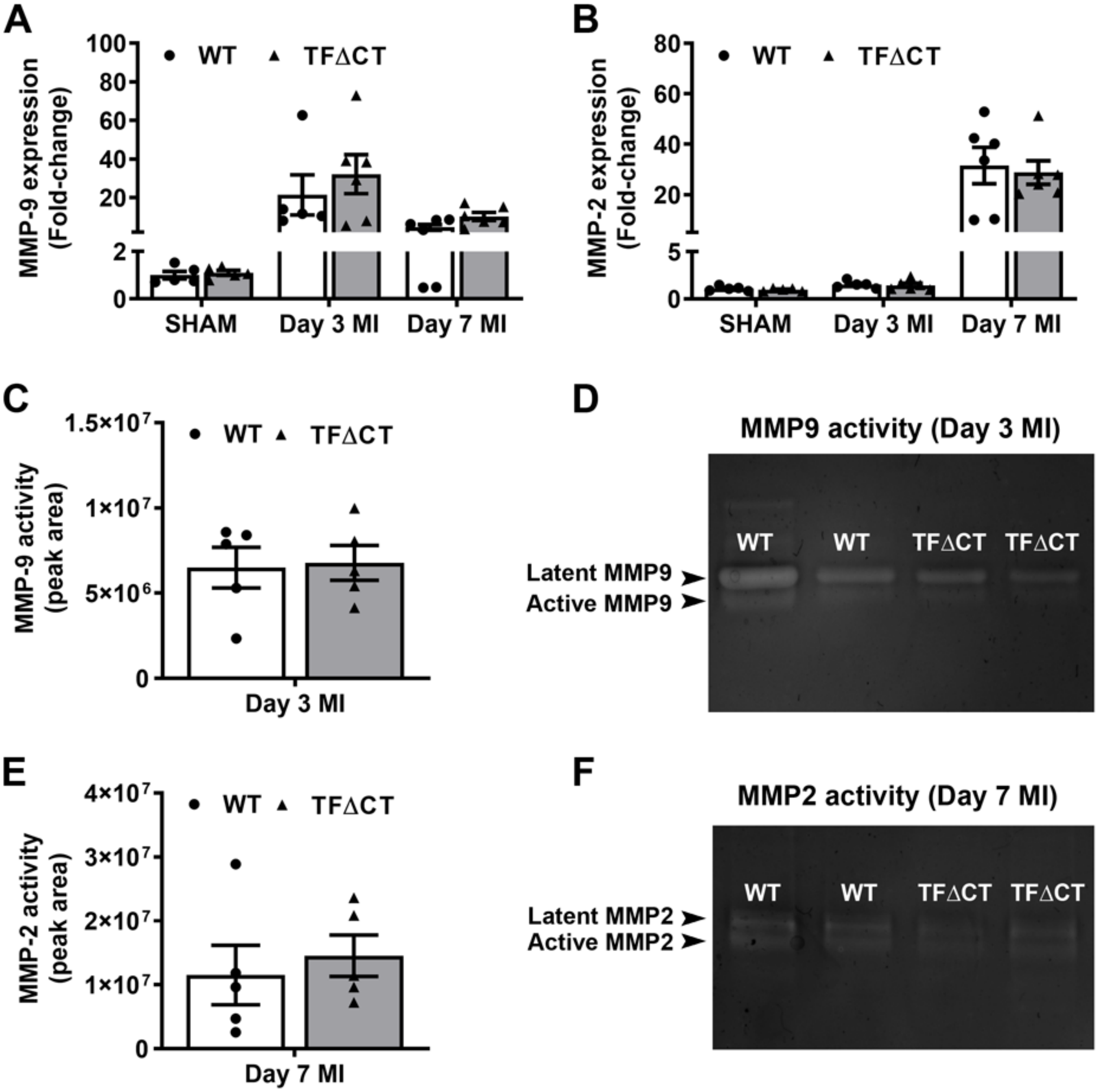

Figure S9. MMP expression and activities following MI. mRNA expression of MMP9 (**A**) and MMP2 (**B**) in the heart tissue (n=5-6 per genotype per time-point). **C,** MMP9 activity at 3 days post-MI is quantified by peak areas as described in the methods. **D,** A representative image of zymography of MMP9 in the infarcted heart tissue. Mann–Whitney U test was used for comparison with WT mice. **E,** MMP2 activity at 7 days post-MI is quantified by peak areas as described in the Methods. **F,** A representative image of zymography of MMP2 in the infarcted heart tissue.

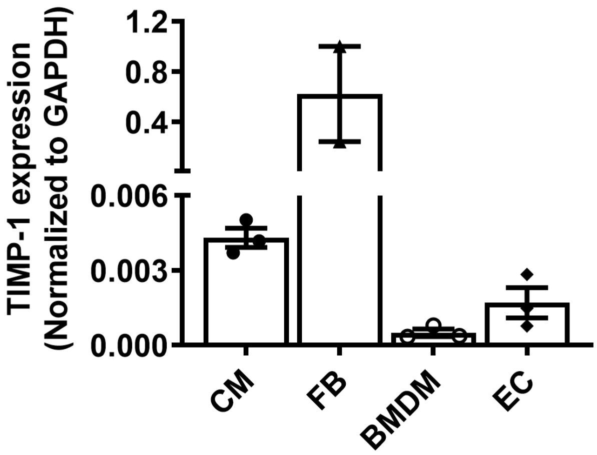

Figure S10. Cell specific gene expression of tissue inhibitor of metalloproteinase 1 (TIMP-1). mRNA was determined by qPCR and normalized to GAPDH. Cells were isolated from 2 - 3 mice per genotype. CM, cardiomyocyte; FB, fibroblast; BMDM, bone marrow derived macrophage; EC, endothelial cell.

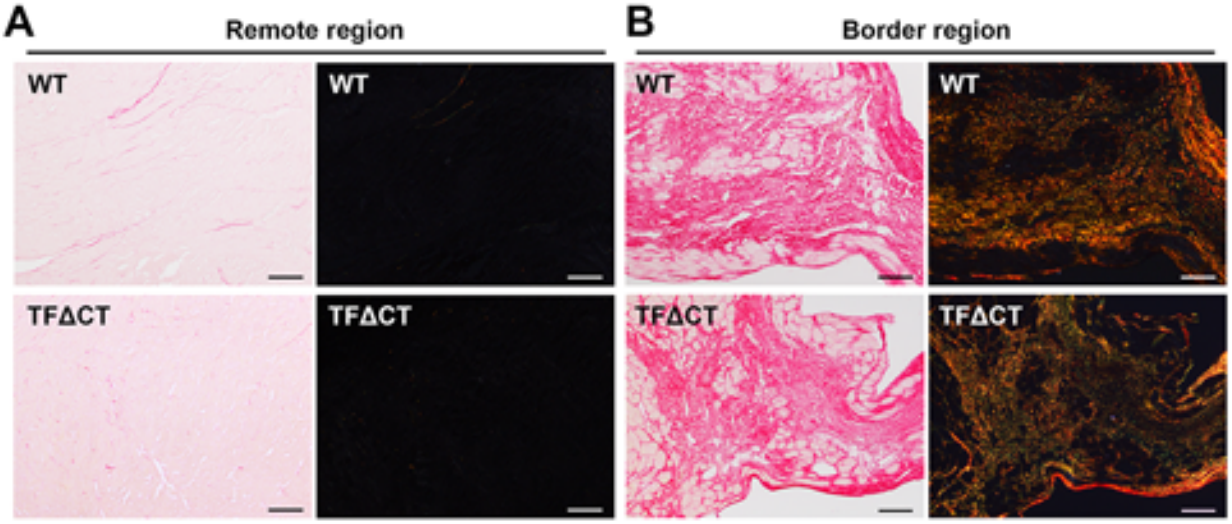

Figure S11. Representative heart sections (28 days post-MI) stained with Picrosirius Red. Collagen deposition in the remote (**A**) and infarct border (**B**) regions. Images were taken under white light or polarized light. Scale bars represent 100 µM.

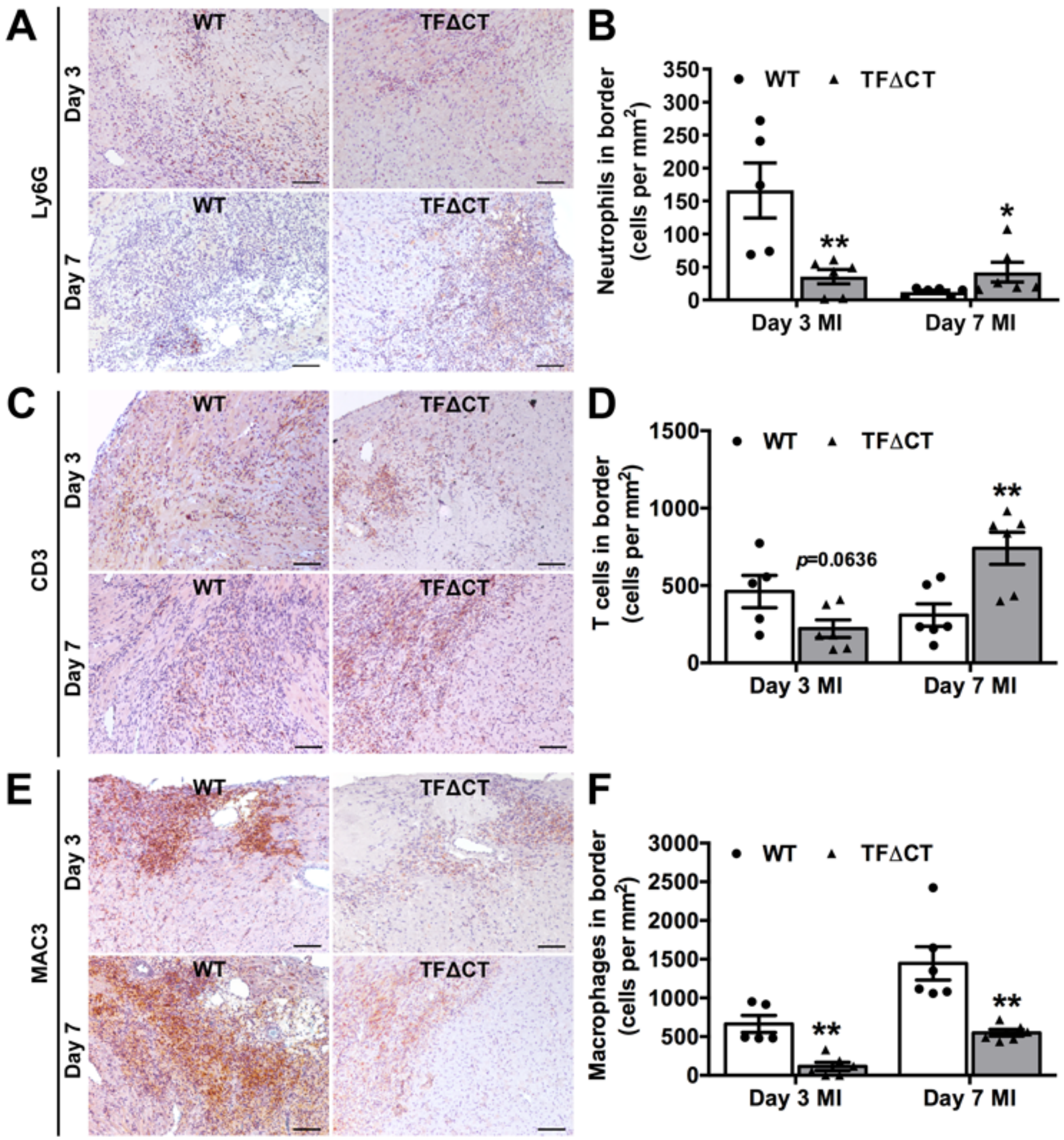

Figure S12. Infiltration of immune cells in the infarct border. A, C, E, Representative images of infarcted myocardium sections stained for neutrophils (A), T cells (C) and macrophages (E) in WT and TFΔCT mice (10x magnification). Scale bar represents 100 µm. **B, D, F,** Quantification of infiltrated neutrophils (B), T-cells (D) and macrophages (F) in the infarct border. N = 5 - 6 per timepoint per genotype; Mann–Whitney U test, *$p < 0.05$ and **$p < 0.01$ compared with WT mice.

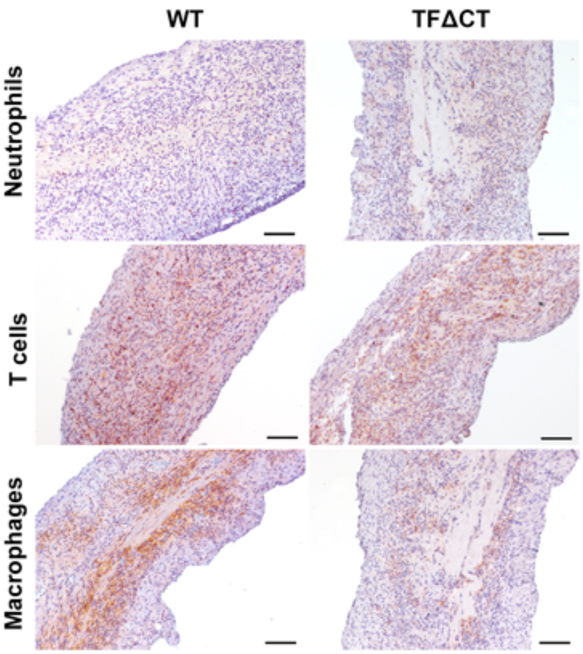

Figure S13. Infiltration of immune cells in the infarct region (an extension of **Figure 5).** Representative images of infarcted myocardium sections at 7 days post-MI stained for neutrophils, T cells and macrophages in WT and TFΔCT mice (10x magnification). Cell specific markers: Ly6G for neutrophils, CD3 for T-cells and MAC3 for macrophages. Scale bar represents 100 µm.

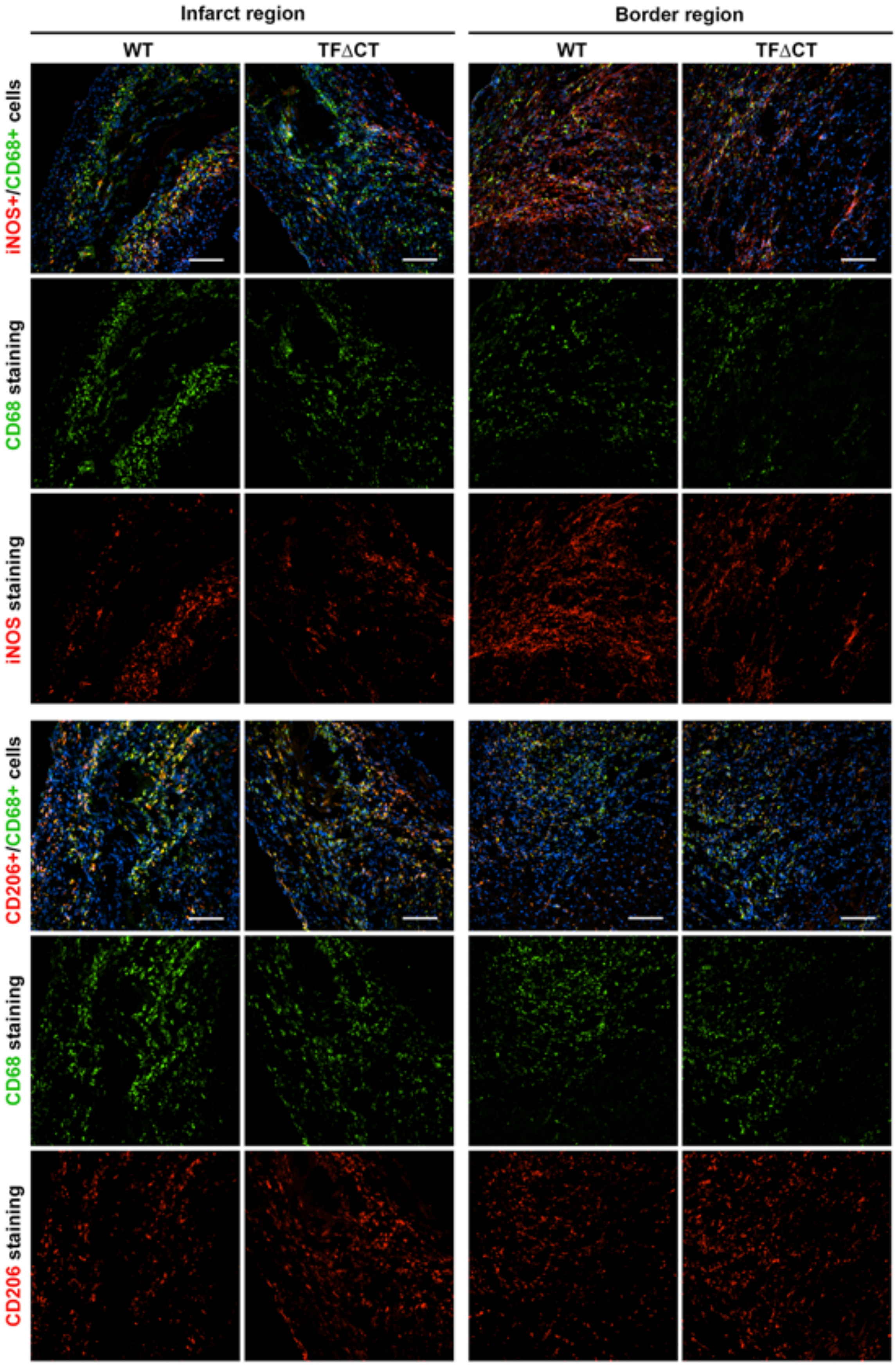

Infarct region
Border region
WT
TFΔCT
WT
TFΔCT
iNOS+/CD68+ cells
CD68 staining
iNOS staining
CD206+/CD68+ cells
CD68 staining
CD206 staining

Figure S14. Proinflammatory and reparative macrophages in MI heart.
Immunofluorescence staining of macrophages in both WT and TFΔCT hearts at day 7 post-MI. $CD68^{+}$ macrophages expressing iNOS (a marker for pro-inflammatory macrophage subtype-M1) or CD206 (a marker for reparative macrophage subtype-M2) in both infarct and border region. Scale bars = 100 μM.

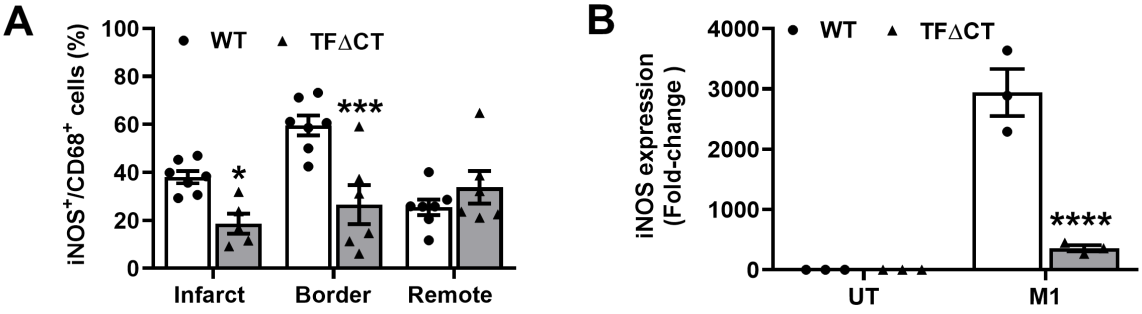

Figure S15. **Macrophage polarization. A,** Percentage M1 macrophages (CD68+iNOS+) in different area of murine heart tissue, WT and TFΔCT and at day 7 after MI. **B,** iNOS gene expression in BMDM of WT and TFΔCT mice, polarized with LPS and IFN-ɣ to M1 pro-inflammatory phenotype. $*P < 0.05$, $***p < 0.001$, $****p < 0.0001$ compared with WT mice by two-way ANOVA with Bonferroni *post hoc* test, n = 3 - 6 per genotype. BMDM, bone marrow derived macrophages.

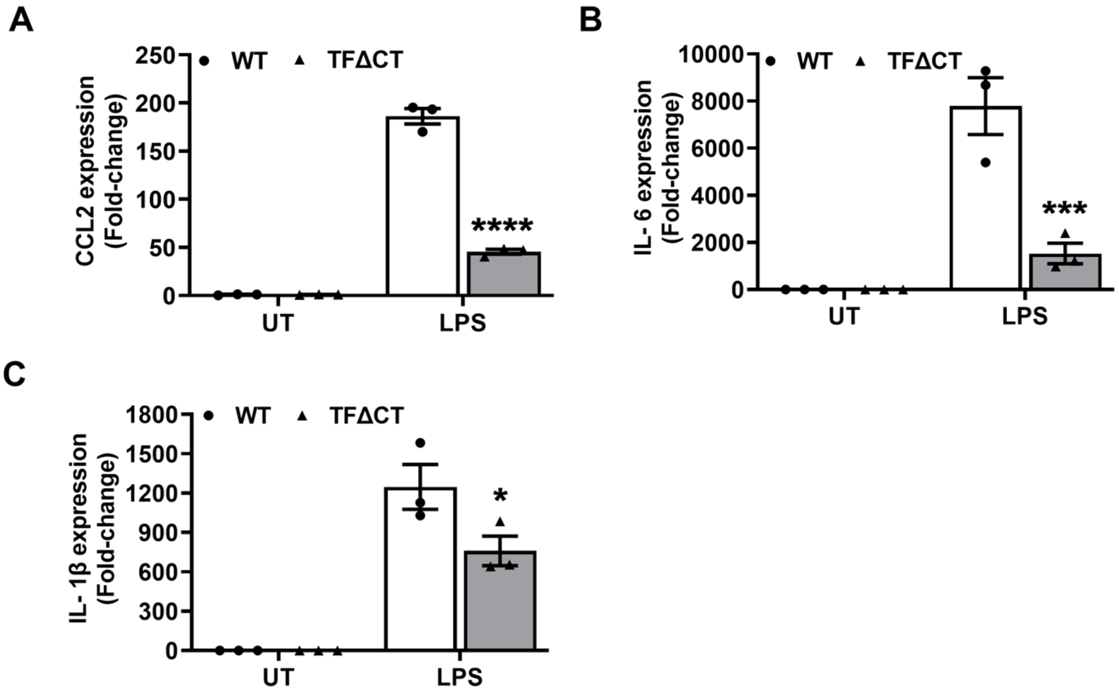

Figure S16. Lack of the TF cytoplasmic domain inhibits expression of pro-inflammatory cytokines in BMDM. BMDM from WT or TFΔCT mice were stimulated with 10 ng/ml LPS. Expression of (**A**) CCL2, (**B**) IL-6, and (**C**) IL-1β in BMDM was determined by qPCR and presented as fold-changes to the mRNA levels in untreated cells (UT). *$P < 0.05$, ***$p < 0.001$, ****$p < 0.0001$ compared with WT BMDM by two-way ANOVA with Bonferroni *post hoc* test; bars represent mean ± SEM of three independent experiments. BMDM, bone marrow derived macrophages.

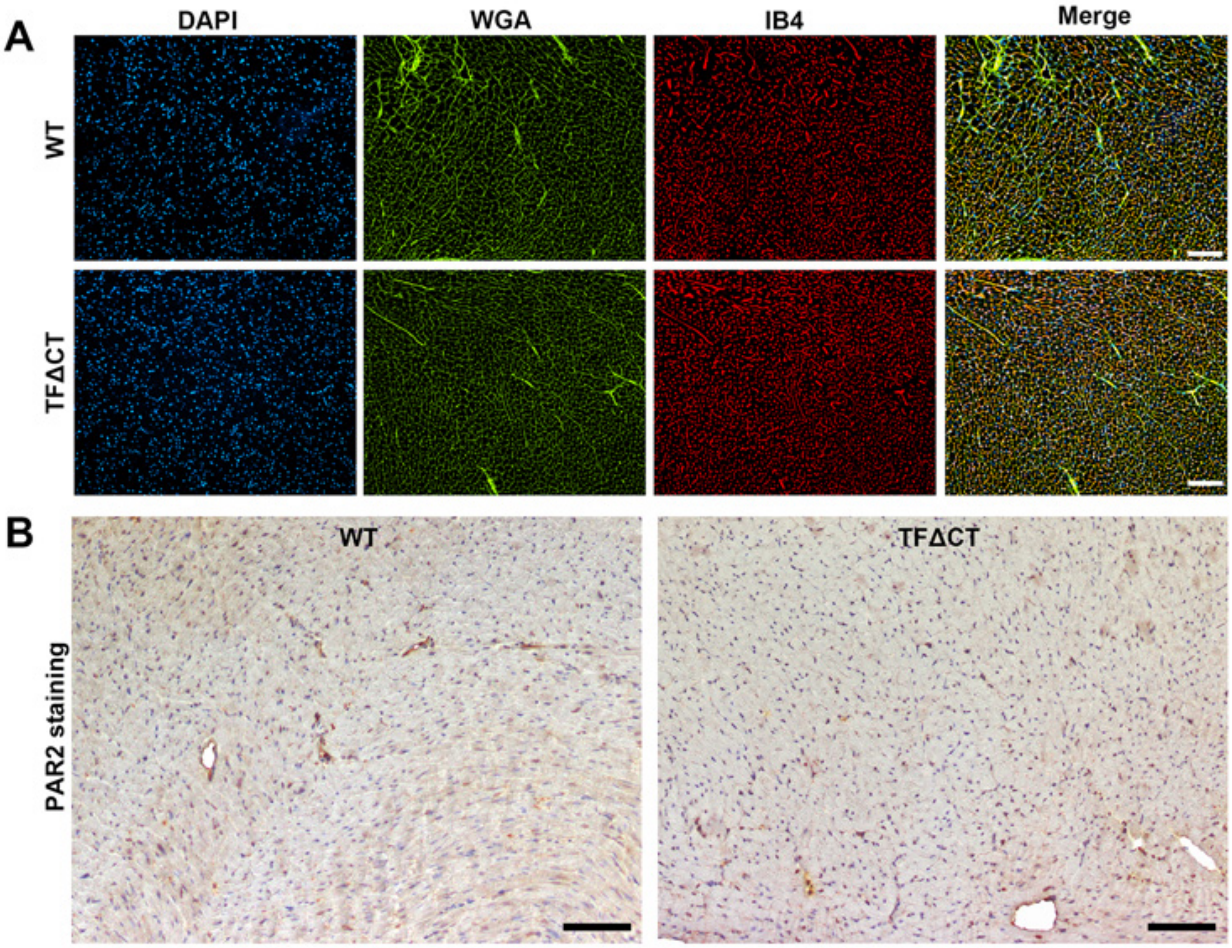

Figure S17. **Baseline blood vessels and PAR2 expression in the heart. A,** Representative images of sham-operated mouse heart sections stained with DAPI (blue), WGA (green) and IB4 (red). **B,** Representative sham-operated mouse heart sections stained for PAR2. Scale bars represent 100 μM.

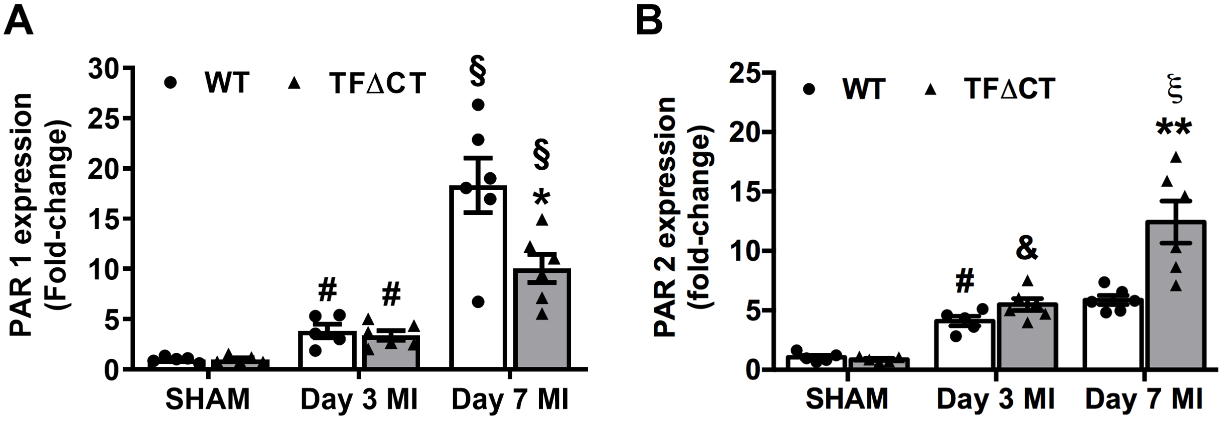

Figure S18. The TF Cytoplasmic Domain Differentially Regulates PAR1 and PAR2 Expression in MI. mRNA levels of PAR1 (**A**) and PAR2 (**B**) were determined in infarcted heart tissue at 3 and 7 days post-MI. The mRNA level was normalized to GAPDH and presented relative to a genotype match sham set as 1 (n = 5 - 6 per genotype per time-point). Mann–Whitney U test, *$p < 0.05$, **$p < 0.01$ compared with WT mice; #$p < 0.01$, &$p < 0.001$ compared with sham; ξ$p < 0.05$, §$p < 0.01$ compared day 3 post-MI.

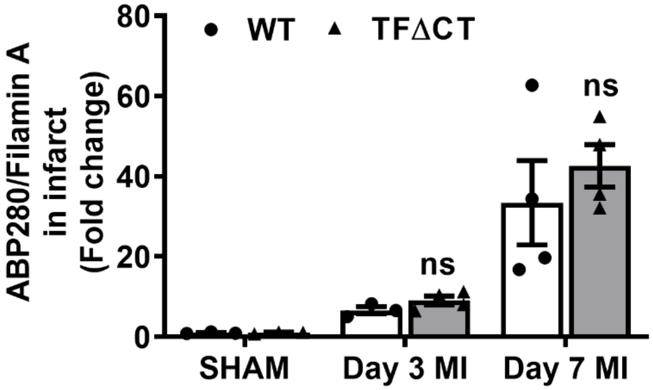

Figure S19. Expression of ABP280/Filamin A in MI. mRNA levels of ABP280/Filamin A were determined in infarcted heart tissue at 3 and 7 days post-MI. The mRNA level was normalized to GAPDH and presented as fold-change of SHAM. Data were analyzed using two-way ANOVA with Bonferroni *post hoc* test; n = 3 - 4 mice per genotype per time point.

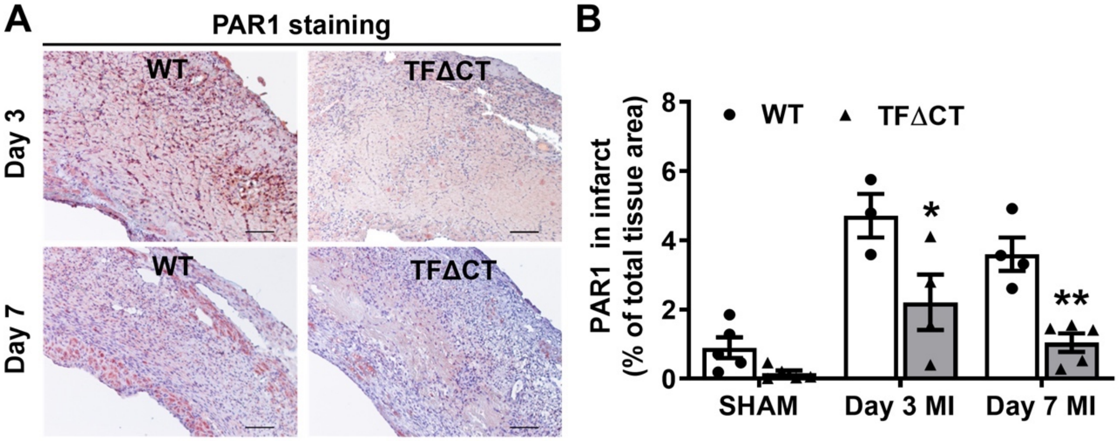

Figure S20. PAR1 protein expression in infarcted heart tissue. A, Representative images of PAR1 staining in the infarct region at 3 and 7 days post-MI in WT and TFΔCT mice (10x magnification). Scale bars represent 100 µm. **B,** Quantification of PAR1 staining. $*P < 0.05$; $**p < 0.01$ compared to WT. Two-way ANOVA with Bonferroni *post hoc* test; n = 3-5 mice per genotype per time-point.

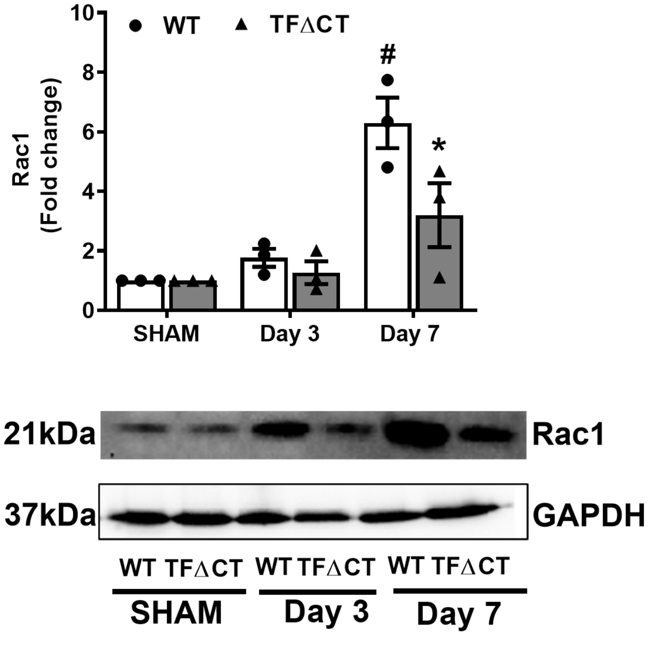

Figure S21. Expression of Rac1 in the infarcted myocardium. Relative expression of Rac1 was determined by Western blot and normalized to GAPDH (n = 3 mice per genotype per time-point; GAPDH as the loading control). Data are presented as fold change of respective sham and reported as mean ± SEM, $*p < 0.05$ compared with WT mice; $\#p < 0.05$, Mann–Whitney U test compared with sham.

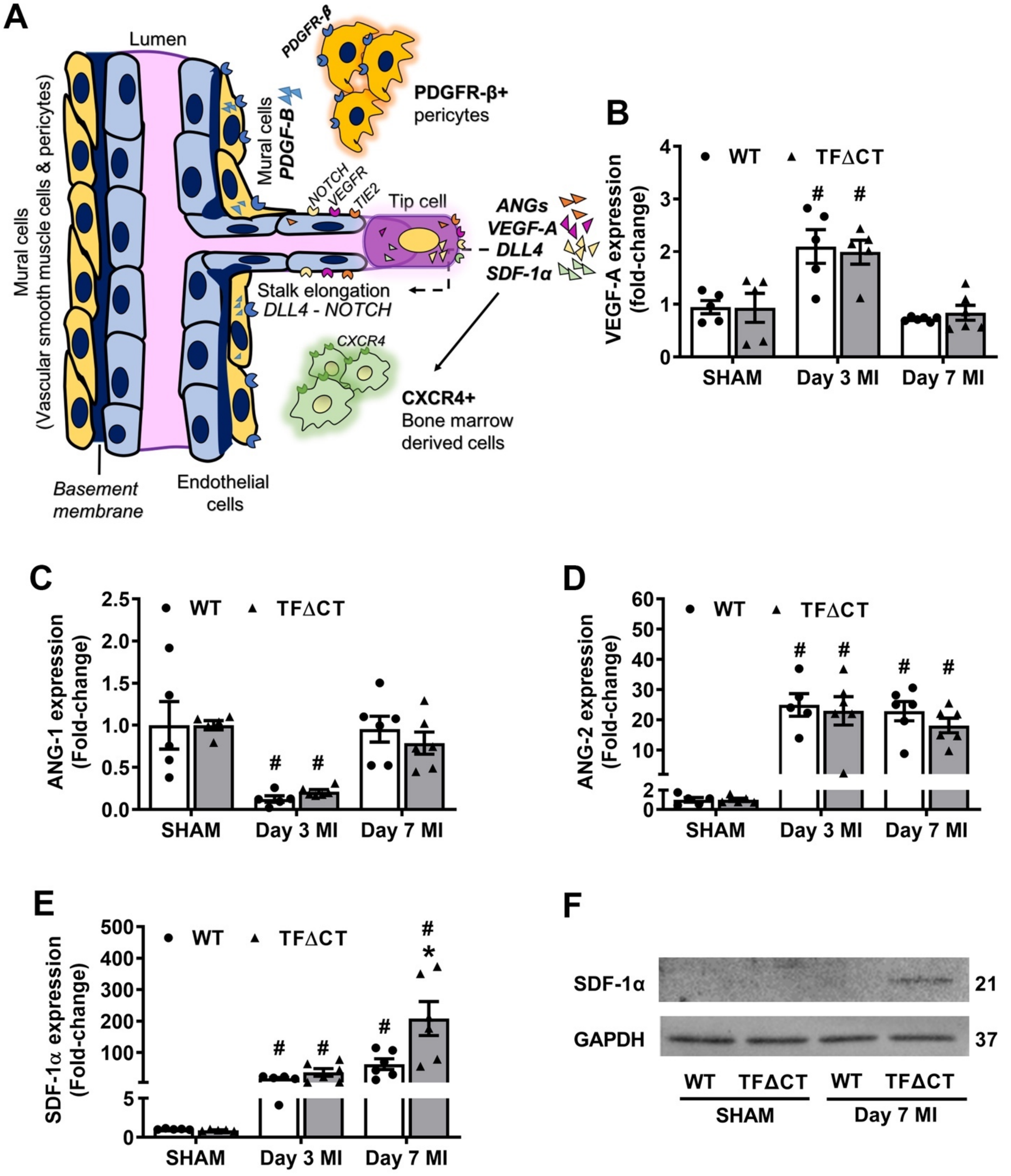

Figure S22. The TF cytoplasmic domain regulates angiogenic signaling pathways in the infarcted heart (extension of Figure 8). A, A schematic diagram illustrating key angiogenic signaling molecules examined in the infarcted heart. **B-E,** mRNA expression levels of VEGF-A, angiopoietin 1 and 2, and SDF-1α in the infarcted myocardium. Relative mRNA expression was

normalized to sham-operated mice of its own genotype. N = 5 - 6 per genotype per time-point. Mann–Whitney U test, **p* < 0.05 compared with WT mice; #*p* < 0.05 compared with sham. **F**, Western blot analysis of SDF-1α protein expression in infarcted myocardium at day 7 post MI. VEGF-A, vascular endothelial growth factor A; ANGs, angiopoietin 1 and 2. SDF-1α, stromal cell-derived factor 1.

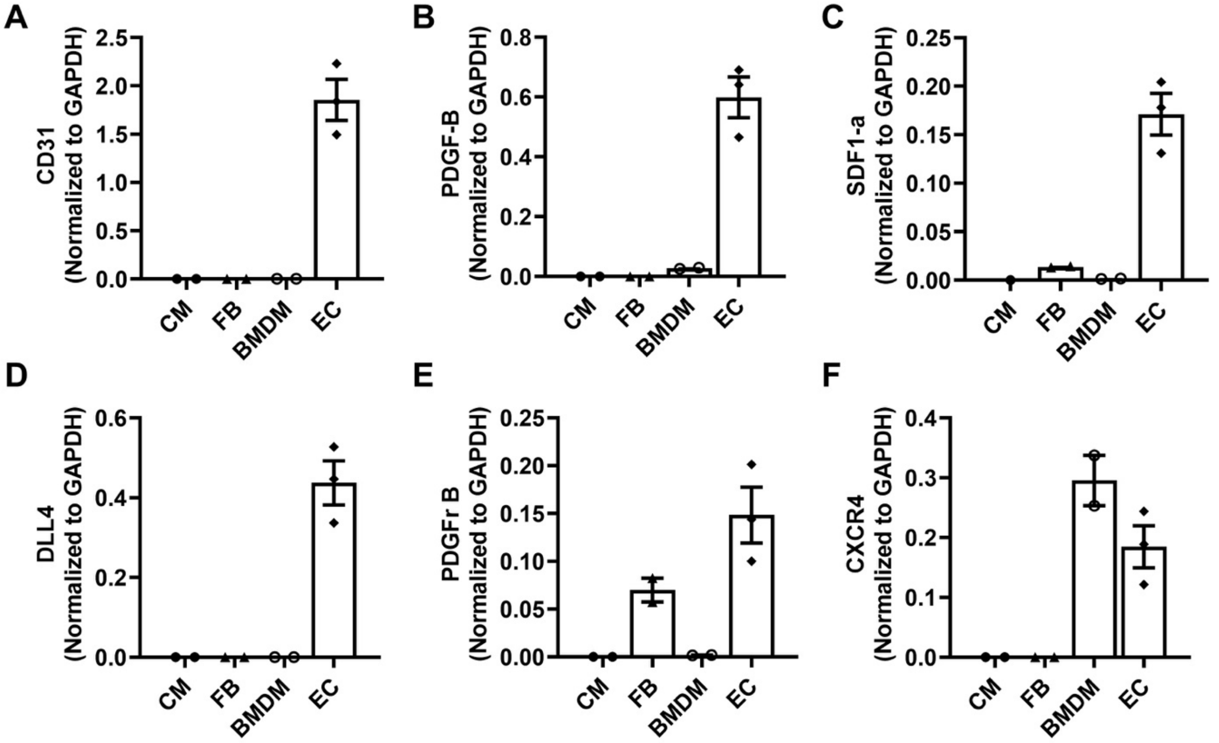

Figure S23. Cell specific expression of angiogenic factors in the heart. Cardiac cells were isolated by FACS sorting and BMDM were differentiated as described in the Methods. **A,** Purity of isolated cells was exampled by the specific expression of CD31 in isolated endothelial cells. **B-F,** mRNA expression of various angiogenic factors, platelet-derived growth factor B (PDGF-B), platelet-derived growth factor receptor β (PDGFR-β), Delta Like Canonical Notch Ligand 4 (DLL4), stromal cell-derived factor-1 (SDF-1α) and C-X-C chemokine receptor type 4 (CXCR4), was determined by qPCR and normalized to GAPDH. Cells were isolated from 2 - 3 WT mice. CM, cardiomyocyte; FB, fibroblast; BMDM, bone marrow derived macrophage; EC, endothelial cell.

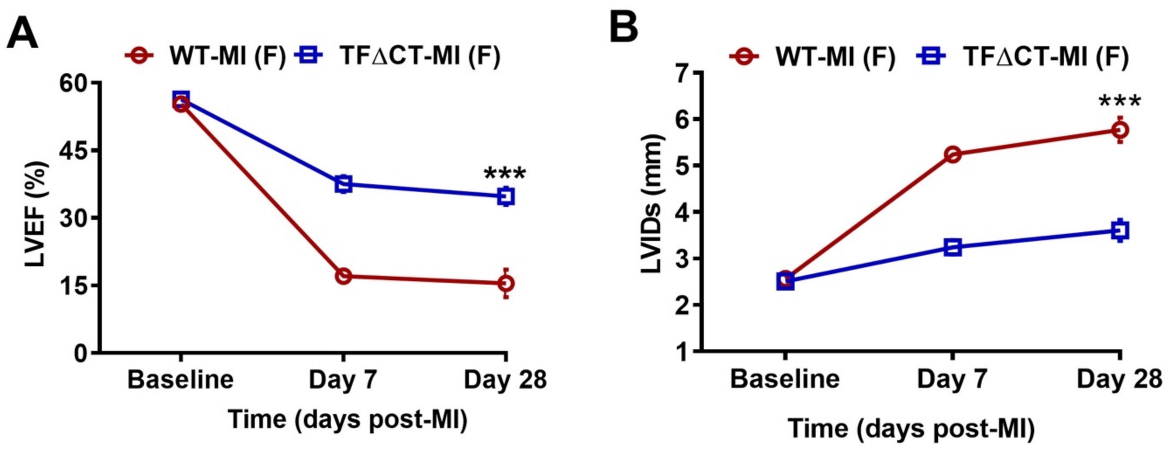

Figure S24. Lack of the TF cytoplasmic domain protects cardiac function in female mice after MI. LVEF and LVIDs were determined by echocardiography. ****P* < 0.001 compared with WT mice by two-way ANOVA with Bonferroni *post hoc* test; n = 3 for WT mice and n = 5 for TFΔCT mice.

Videos I - IV:

B-mode cine loop in long-axis view with semiautomatically traced endocardial and epicardial borders (heart shape; in green) with vector arrows (orange) represent myocardial contraction and relaxation during three cycles at baseline in WT (**Video I. Baseline_WT**) and TFΔCT (**Video II. Baseline_TFΔCT**) mice; and at day 28 after MI in WT (**Video III. MI_WT**) and TFΔCT (**Video IV. MI_ TFΔCT**) mice.

SUPPLEMENTAL TABLES

Table S1. List of Antibodies used in present study

Marker	Application	Dilution	Suppliers and cat. #
Primary antibodies			
Fluorescein labeled Wheat Germ Agglutinin (WGA-FITC)	Microscopy	1:100	Vector Lab (FL-1021)
Biotinylated Griffonia Simplicifolia Lectin I (GSL I) isolectin B4	Microscopy	1:25	Vector Lab (B-1205)
PAR-1	Microscopy	1:200	ThermoFisher Scientific (BS-0828R)
PAR2	Microscopy	1:500	ThermoFisher Scientific (PA5-77685)
CD142 (TF)	Microscopy	1:100	ThermoFisher Scientific (PA5-47465)
aSMA	Microscopy	1:1000	Abcam (ab124964)
Ki67	Microscopy	1:100	ThermoFisher Scientific (14-5698-82)
CD3	Microscopy	1:100	Dako (DKO.A045229)
Ly-6G	Microscopy	1:100	BD Pharmingen (551459)
MAC3	Microscopy	1:100	BD Pharmingen (553322)
CD68	Microscopy	1:200	Bio-Rad (MCA 1957)
Vimentin	Microscopy	1:500	Abcam (ab45939)
TNNT2	Microscopy	1:500	ThermoFisher Scientific (MS-295-P1)
iNOS	Microscopy	1:500	Abcam (ab15323)
CD206	Microscopy	1:200	Bio-Rad (MCA2235)
Rac1	Immunoblot	1:2000	EMD Millipore (05-389)
GAPDH	Immunoblot	1:10000	Abcam (EPR16891)
DLL4	Immunoblot	1:1000	Abcam (ab183532)
SDF-1α	Immunoblot	1:1000	Torrey Pines Biolabs (TP201)
CXCR4	Immunoblot	1:1000	ThermoFisher Scientific (OPA1-15093)
PDGF-B	Immunoblot	1:5000	Abcam (ab178409)
PDGFR-β	Immunoblot	1:1000	Merk (06-498-I)
CD45.1 – PerCP-Cy5.5	Flow Cytometry	1:200	eBioscience (Clone A20) (45-0453-82)
CD45.2 - FITC	Flow Cytometry	1:200	eBioscience (Clone 104)

			(11-0454-82)
CD45 – Alexa Fluor® 700	FACS	1:400	Biolegend<br>(Clone 30-F11 (RUO))<br>(103128)
CD31 - PE-CY7	FACS	1:200	Biolegend<br>(Clone 390)<br>(102524)
Podoplanin – APC-Cy7	FACS	1:200	Biolegend<br>(Clone 8.1.1)<br>(127418)
Isotype Controls			
Mouse IgG1 Isotype Control	Microscopy	1:500	ThermoFisher Scientific (02-6100)
Goat IgG Isotype Control	Microscopy	1:500	Abcam (ab37373)
Rat IgG Isotype	Microscopy	1:500	Abcam (ab37361)
Rabbit IgG Isotype	Microscopy	1:500	Abcam (ab172730)
Isotype Ctrl Antibody - PerCP-Cy5.5 Mouse IgG2a,κappa	Flow Cytometry	1:200	Biolegend,<br>(Clone MOPC-173)<br>(400251)
Isotype Ctrl Antibody FITC, Mouse IgG2a,kappa	Flow Cytometry	1:200	eBioscience<br>(Clone eBM2a)<br>(11-4724-81)
Secondary antibodies			
F(ab')2-Goat anti-Rat IgG(H+L) Cross-Adsorbed Secondary Antibody, HRP	Microscopy	1:500	ThermoFisher Scientific (A24549)
Streptavidin, Alexa Fluor 568 conjugate	Microscopy	1:1000	ThermoFisher Scientific (S11226)
Donkey anti-Rabbit IgG (H+L) Highly Cross-Adsorbed Secondary Antibody, Alexa Fluor 488	Microscopy	1:1000	ThermoFisher Scientific (A-21206)
Donkey anti-Rabbit IgG (H+L) Highly Cross-Adsorbed Secondary Antibody, Alexa Fluor 594	Microscopy	1:1000	ThermoFisher Scientific (A-21207)
Donkey anti-Rat IgG (H+L) Highly Cross-Adsorbed Secondary Antibody, Alexa Fluor 488	Microscopy	1:1000	ThermoFisher Scientific (A-21208)
Donket anti Goat-IgG (H+L), Highly Cross-	Microscopy	1:1000	ThermoFisher Scientific (A-11058)

Absorbed Secondary Antibody, Alexa Fluor 594			
Donkey anti-Mouse IgG (H+L) Secondary Antibody, Alexa Fluor 488	Microscopy	1:1000	ThermoFisher Scientific (A-21203)
Goat anti-Rat IgG (H+L) Highly Cross-Adsorbed Secondary Antibody, Alexa Fluor 647	Microscopy	1:1000	ThermoFisher Scientific (A21247)
Goat anti rabbit-IgG H&L-HRP conjugated	Immunoblot	1:10000	Abcam (ab6721)
Mouse anti-mouse HRP	Immunoblot	1:15000	SuntaCruz (sc-2005)
Extra dyes			
DAPI	Microscopy	1:1000	ThermoFisher (62248)
Mayer's Hematoxylin solution	Microscopy	Neat	Merck (105175)

Table S2. List of primers used for RT-PCR

	Gene	Forward primer	Reverse primer
Murine	**ANG-1**	5’-CATTCTTCGCTGCCATTCTG-3’	5’-GCACATTGCCCATGTTGAATC-3’
	**ANG-2**	5’-CAGCCA CGGTCAACAACTC-3’	5’-CTTCTTTACGGATAGCAACCGAG-3’
	**COL1A1**	5'-TCAAGGTCTACTGCAACATGG-3'	5'-AATCCATCGGTCATGCTCTCT-3'
	**COL3A1**	5'-GATGCCATTAGAGCCACGTT-3'	5'-AAGAGTGGTGACAGAGGAGAA-3'
	**CXCR4**	5’-CTGCCCACCATCTACTTCATC-3’	5’-CGTCATGCTCCTTAGCTTCTT-3’
	**DLL4**	5’-GGGAACAGAGTTGAGGAGTTAG-3’	5’-CACTCTCTGGAGAACAGTCAAG-3’
	**GAPDH**	5'-GTGGAGTCATACTGGAACATGTAG-3'	5'-AATGGTGAAGGTCGGTGTG-3'
	**VEGF-A**	5'-TGGTGACATGGTTAATCGGT-3'	5'-AGAAAGACAGAACAAAGCCAGA-3'
	**MMP-2**	5-’AACTACGATGATGACCGGAAGTG-3’	5’-TGGCATGGCCGAACTCA-3’
	**MMP-9**	5’-CGAACTTCGACACTGACAAGAAGT-3’	5’-GCACGCTGGAATGATCTAAGC-3’
	**PAR 1**	5’-TGAACCCCCGCTCATTCTTTC-3’	5’-CCAGCAGGACGCTTTCATTTTT-3’
	**PAR 2**	5’- GAGTAGGGCTCCGAGTTTCG-3’	5’- CGCCAGAAGGGTGATACCTC-3’
	**PDGF-B**	5’-CACACACCTTCTCTGATGGATTA-3’	5’-CACAGAGTGGAGGTAGAGAAATG-3’
	**PDGFR-B**	5’-TCCCACATTCCTTGCCCTTC-3’	5’-GCACAGGGTCCACGTAGATG-3’
	**SDF-1A**	5’-ACAAGTGTGCATTGACCCGA-3’	5’-GCATCTCCCACGGATGTCAG-3’
	**Total TF**	5'-AACCCACCAACTATACCTACACT-3'	5'-GTCTGTGAGGTCGCACTCG-3'
	**TGFβ-1**	5'-TGTTGGTTGTAGAGGGCAAG-3'	5'-CTACCATGCCAACTTCTGTCT-3'
	**TIMP-1**	5'-TCTGGCATCTGGCATCCTCTTG-3'	5'-AACGCTGGTATAAGGTGGTCTCG-3'
	**TSP-1**	5'-TGGCCAGCGTTGCCA-3'	5'-TCTGCAGCACCCCCTGAA-3'
	**OPN**	5'-CTCCATCGTCATCATCATCG-3'	5'-TGCACCCAGATCCTATAGCC-3'

Table S3. Echocardiography characterization of BM transplanted mice.

	WT/WT BM	*WT/TFΔCT BM*	*TFΔCT/WT BM*	*TFΔCT/ TFΔCT BM*
**HR (bpm)**	454.4 ± 23.3	427.4 ± 25.3	406.4 ± 7.6	420.6 ± 7.7
**ESV (µL)**	91.4 ± 5.0§	66.7 ± 4.8*	73.6 ± 5.7	69.9 ± 4.1*
**EDV (µL)**	112.6 ± 4.8	95.8 ± 5.2	99.3 ± 6.8	98.9 ± 5.4
**EF (%)**	19.1 ± 1.4#	30.7 ± 1.7***	26.1 ± 1.8*	29.4 ± 0.7***
**FS (%)**	8.2 ± 0.9§	14.9 ± 1.3***	12.8 ± 0.9*	13.8 ± 0.4**
**LVIDs (mm)**	4.6 ± 0.2	4.1 ± 0.2	4.1 ± 0.2	4.3 ± 0.1
**LVIDd (mm)**	5.0 ± 0.2	4.8 ± 0.1	4.7 ± 0.1	4.9 ± 0.1

Echocardiography was performed on the mice at 28 days after MI: WT/WT BM, WT mice receiving WT BM (n = 7); WT/TFΔCT BM, WT mice receiving TFΔCT BM (n = 8); TFΔCT/WT BM, TFΔCT mice receiving WT BM (n = 8); TFΔCT/TFΔCT BM, TFΔCT mice receiving TFΔCT mice (n = 7). One-way ANOVA followed by Bonferroni *post hoc* testing of multi-group comparisons. Compared with WT/WT BM mice: *$p < 0.05$; **$p < 0.01$; ***$p < 0.001$. Compared with WT/TFΔCT BM mice: §$p < 0.05$; #$p < 0.001$. HR, heart rate (beat per minute); ESV, end systolic volume; EDV, end diastolic volume; EF, ejection fraction; FS, fractional shortening; LVIDs, left ventricular internal diameter end systole; LVIDd, left ventricular internal diameter end diastole.

Table S4. Cytokines and Chemokines expression in remote and infarcted heart tissue following MI.

	Day 3 post MI				Day 7 post MI				SHAM	
	Infarct		Remote		Infarct		Remote			
	WT	*TFΔCT*	*WT*	*TFΔCT*	*WT*	*TFΔCT*	*WT*	*TFΔCT*	*WT*	*TFΔCT*
**IL-1α**	1702.8 ± 759.5	652.8 ± 147.8	224.3 ± 63.5	264.3 ± 27.6	182.3 ± 24.4	164.0 ± 24.8	259.26 ± 69.0	233.4 ± 28.0	246.6 ± 29.9	140.9 ± 35.3
**IL-1β**	**87.5 ± 21.0**	**31.5 ± 6.7****	40.1 ± 10.8	41.6 ± 7.7	**76.7 ± 11.7**	**24.1 ± 5.7***	50.14 ± 10.3	39.9 ± 10.7	25.7 ± 7.2	39.2 ± 2.41
**IL-2**	2939.2 ± 1231.8	1425.4 ± 340.1	461.0 ± 139.2	528.7 ± 50.2	339.4 ± 55.9	366.6 ± 62.6	528.4 ± 133.8	489.9 ± 96.7	496.6 ± 15.2	266.7 ± 56.9
**IL-3**	**46.6 ± 20.3**	**8.1 ± 3.0***	6.1 ± 0.9	8.8 ± 1.6	16.6 ± 3.6	8.8 ± 2.5	7.7 ± 0.9	10.2 ± 1.8	2.68 ± 0.8	10.4 ± 1.2
**IL-4**	**250.0 ± 119.9**	**32.4 ± 10.9***	12.6 ± 3.7	12.9 ± 2.4	80.2 ± 13.9	36.5 ± 13.9	11.1 ± 2.3	12.9 ± 2.8	11.0 ± 0.74	13.2 ± 2.6
**IL-5**	125 ± 38.5	41.3 ± 7.5	32.0 ± 12.7	28.1 ± 4.3	32.7 ± 8.6	30.3 ± 6.6	47.7 ± 11.4	30.8 ± 5.1	25.8 ± 22.8	28.1 ± 4.0
**IL-6**	**338.5 ± 120.2**	**98.4 ± 21.7***	47.5 ± 11.9	35.0 ± 2.5	56.4 ± 4.8	58.3 ± 4.7	45.7 ± 10.7	32.7 ± 4.1	60.5 ± 6.1	24.2 ± 1.0
**IL-9**	292.4 ± 101.8	150.5 ± 29.1	133.1 ± 31.0	145.5 ± 20.3	80.4 ± 5.9	73.3 ± 10.1	162.9 ± 33.7	138.8 ± 24.9	154.7 ± 35.2	108.0 ± 20.9
**IL-10**	459.5 ± 188.9	129.7 ± 32.5	77.6 ± 9.4	108.7 ± 17.4	252.2 ± 39.7	146.5 ± 38.2	89.2 ± 10.0	119.1 ± 21.0	28.4 ± 9.6	101.4 ± 14.01
**IL-12(p40)**	595.1 ± 274.3	174.3 ± 23.3	53.9 ± 9.1	71.1 ± 9.6	371.6 ± 23.1	347.9 ± 64.4	63.1 ± 2.5	74.4 ± 17.9	25.1 ± 3.5	61.5 ± 5.6
**IL-12(p70)**	**3246.3 ± 1519.7**	**770.5 ± 127.5***	335.7 ± 67.0	532.0 ± 46.2	1522.6 ± 276.3	1105.9 ± 255.1	405.8 ± 60.9	532.0 ± 60.9	210.1 ± 40.9	365.9 ± 20.8
**IL-13**	2038.4 ± 890.4	554.1 ± 191.3	538.5 ± 69.8	514.8 ± 69.8	870.5 ± 131.8	723.2 ± 184.9	618.3 ± 56.3	520.4 ± 74.4	391.6 ± 176.7	719.4 ± 134.7
**IL-17**	189.6 ± 100.5	52.0 ± 11.1	25.5 ± 4.9	39.0 ± 5.3	97.8 ± 16.3	67.9 ± 18.9	29.0 ± 2.9	37.9 ± 5.6	9.7 ± 1.7	35.4 ± 4.6
**TNFα**	**1142.4 ± 396.3**	**327.5 ± 48.6****	207.8 ± 66.5	138.8 ± 18.4	561.1 ± 39.4	466.1 ± 98.8	267.4 ± 54.4	145.5 ± 25.6	329.8 ± 281.0	181.6 ± 1.68
**IFN-γ**	**477.9 ± 205.9**	**155.8 ± 39.0***	145.5 ± 23.5	158.4 ± 22.4	190.9 ± 20.2	150.8 ± 26.9	158.3 ± 25.2	141.5 ± 19.3	144.7 ± 36.9	141.4 ± 30.6
**G-CSF**	**173.5 ± 93.8**	**28.8 ± 3.4***	11.9 ± 2.9	18.8 ± 5.1	75.7 ± 17.5	34.0 ± 6.7	16.7 ± 1.7	17.6 ± 4.2	9.0 ± 1.19	18.6 ± 4.7
**GMCSF**	721.4 ± 430.7	165.5 ± 29.3	65.4 ± 10.5	77.7 ± 14.0	**254.2 ± 63.1**	**103.4 ± 33.8***	68.3 ± 5.7	79.6 ± 18.5	39.2 ± 13.2	67.1 ± 7.8
**CXCL-1 (KC)**	586.7 ± 177.8	228.7 ± 21.6	55.7 ± 8.4	61.6 ± 9.5	216.7 ± 37.8	185.0 ± 28.2	47.5 ± 3.0	57.3 ± 8.8	18.9 ± 4.5	61.7 ± 0.8
**CCL2 (MCP-1)**	4169.3 ± 513.4	3392.6 ± 280.0	770.4 ± 131.1	708.5 ± 55.2	2200.3 ± 254.8	2133 ± 241.7	442.1 ± 40.5	510.3 ± 102.0	184.4 ± 10.7	424.0 ± 94.1
**CCL3 (MIP-1α)**	120.2 ± 20.2	99.0 ± 10.8	23.1 ± 3.8	18.3 ± 3.2	**126.2 ± 15.4**	**178.8 ± 9.3****	7.2 ± 1.3	12.3 ± 3.4	2.6 ± 0.2	4.1 ± 0.5
**CCL4 (MIP-1β)**	380.9 ± 85.9	185.8 ± 14.9	152.4 ± 40.0	77.0 ± 6.5	169.9 ± 53.4	241.9 ± 56.1	145.3 ± 46.9	133.1 ± 36.9	27.1 ± 7.5	66.9 ± 4.7
**CCL5 (RANTES)**	1843.2 ± 681.3	749.8 ± 173.5	327.9 ± 81.0	347.0 ± 39.8	619.2 ± 142.9	836.4 ± 129.1	373.9 ± 59.2	317.5 ± 43.9	254.3 ± 48.8	266.2 ± 22.4
**CCL11 (Eotaxin)**	351.3 ± 76.3	237.8 ± 22.1	72.3 ± 8.3	84.7 ± 10.4	111.7 ± 17.5	138.8 ± 16.4	64.2 ± 3.7	69.1 ± 9.9	43.7 ± 18.2	54.3 ± 11.7

Protein concentrations of cytokines and chemokines in lysates of infarcted myocardium were quantified by multiplex assay at different time-points post-MI. N = 5-6 per genotype per time-point; Mann–Whitney U test, *$p < 0.05$, **$p < 0.01$ compared to WT mice.

SUPPLEMENTAL REFERENCES

1. Melis E, Moons L, De Mol M, Herbert JM, Mackman N, Collen D, et al. Targeted deletion of the cytosolic domain of tissue factor in mice does not affect development. Biochem Biophys Res Commun. 2001; 286: 580-6.
2. Allijn IE, Czarny BMS, Wang X, Chong SY, Weiler M, da Silva AE, et al. Liposome encapsulated berberine treatment attenuates cardiac dysfunction after myocardial infarction. J Control Release. 2017; 247: 127-33.
3. de Kleijn DPV, Chong SY, Wang X, Yatim S, Fairhurst AM, Vernooij F, et al. Toll-like receptor 7 deficiency promotes survival and reduces adverse left ventricular remodelling after myocardial infarction. Cardiovasc Res. 2019; 115: 1791-803.
4. Yap L, Wang JW, Moreno-Moral A, Chong LY, Sun Y, Harmston N, et al. In Vivo Generation of Post-infarct Human Cardiac Muscle by Laminin-Promoted Cardiovascular Progenitors. Cell Rep. 2019; 26: 3231-45 e9.
5. Wang JW, Fontes MSC, Wang X, Chong SY, Kessler EL, Zhang YN, et al. Leukocytic Toll-Like Receptor 2 Deficiency Preserves Cardiac Function And Reduces Fibrosis In Sustained Pressure Overload. Sci Rep. 2017; 7: 9193.
6. Hu Z, Li G, Wang JW, Chong SY, Yu D, Wang X, et al. Regulation of Blood Pressure by Targeting CaV1.2-Galectin-1 Protein Interaction. Circulation. 2018; 138: 1431-45.
7. Rich L WP. Collagen and Picrosirius red staining : a polarized light assessment of fibrillar hue and spatial distribution. Brazilian J Microbiol. 2005; 22(2):97-104.
8. Whittaker P, Kloner RA, Boughner DR, Pickering JG. Quantitative assessment of myocardial collagen with picrosirius red staining and circularly polarized light. Basic Res Cardiol. 1994; 89: 397-410.
